# Distinct parafascicular neuronal ensembles mediate the sensory and affective dimensions of trigeminal neuralgia

**DOI:** 10.1172/JCI206862

**Published:** 2026-07-14

**Authors:** Yitian Lu, Yangyang Yi, Jiao Liu, Hao Zhi, Qing Chang, Zihao Huang, Yumeng Chen, Han L. Tan, Yiheng Tu, Yun Wang, Cheng Cen

**Affiliations:** 1 Neuroscience Research Institute and Department of Neurobiology, School of Basic Medical Sciences, Key Laboratory for Neuroscience, Ministry of Education/National Health Commission, Peking University, Beijing, China.; 2 State Key Laboratory of Cognitive Science and Mental Health, Institute of Psychology, Chinese Academy of Sciences, Beijing, China.; 3 Department of Psychology, University of Chinese Academy of Sciences, Beijing, China.; 4 Center of Medical and Health Analysis, Peking University Health Science Center, Beijing, China.; 5 Department of Anatomy and Neurobiology, School of Medicine, University of California, Irvine, Irvine, California, USA.; 6 PKU-IDG/McGovern Institute for Brain Research, Peking University, Beijing, China.

**Keywords:** Cell biology, Neuroscience, Neurological disorders, Pain

## Abstract

Trigeminal neuralgia (TN) is a severe orofacial pain disorder accompanied by anxiety, yet its central mechanisms remain elusive. Analysis of human functional MRI data identified the parafascicular nucleus (PF) as a candidate region. Using a TN mouse model, we uncovered 2 spatially and functionally distinct PF neuronal ensembles that separately encoded sensory and affective dimensions of pain. One population received inhibitory input from GABAergic neurons in the oral spinal trigeminal nucleus (Sp5O) and mediated nociception. The second population, driven by a glutamatergic Sp5O-lateral parabrachial nucleus (lPBN)-PF pathway, encoded pain-related anxiety. The engagement of the anxiety-encoding ensemble lagged behind that of the pain-encoding ensemble, with a shorter delay in females. Single-nucleus RNA sequencing identified *Col25a1* and *Syn2* as markers of the anxiety-encoding ensemble. Notably, this population, localized in the medial PF, formed a reciprocal lPBN-mPF-lPBN excitatory feedback loop that sustained affective pain. These findings positioned PF as a key node linking pain and emotion in TN.

## Abstract 2

Distinct brain circuits independently drive facial pain and anxiety in trigeminal neuralgia, revealing how chronic pain and emotional distress become linked.

## Introduction

Trigeminal neuralgia (TN) is a debilitating neuropathic pain disorder characterized by recurrent, paroxysmal episodes of severe facial pain within the distribution of the trigeminal nerve. Even innocuous light stimuli such as speaking, washing the face, or a breeze can provoke intense, shock-like pain, severely disrupting daily life and basic human functions (1–5). Beyond its physical burden, TN is frequently accompanied by psychiatric comorbidities, notably anxiety and depression, highlighting its profound impact on both somatic and mental health (6). Epidemiological studies indicate that TN occurs more frequently in older populations and is more prevalent among women than men (7, 8). Although current treatments, such as anticonvulsant drugs and surgery interventions, can provide relief, they are often limited by side effects, inconsistent efficacy, and the risk of complications (9–11). These limitations underscore the urgent need to elucidate the central mechanisms that drive TN, particularly those linking sensory and affective symptoms, to inform the development of more effective and targeted therapeutic strategies.

The pathophysiology of TN is thought to involve a multifactorial interplay of anatomical and neurophysiological factors, leading to heightened excitability of nociceptive trigeminal axons, central sensitization, and widespread neural plasticity changes (12). These contribute to the pattern of whole-brain structural and functional abnormalities, including regional gray matter volume loss (13–15). As the first central relay for trigeminal afferents, the spinal trigeminal nuclear complex (Sp5) — composed of the oralis (Sp5O), interpolaris (Sp5I), and caudalis (Sp5C) subnuclei — plays a critical role in transmitting and processing orofacial sensory information. Among them, the caudal subnucleus Sp5C is primarily involved in nociceptive transmission, receiving inputs from small-diameter Aδ and C fibers that encode facial pain and thermal stimuli, while the oral subnucleus Sp5O mainly processes low-threshold Aβ/Aδ fiber mechanosensory and intraoral information (16, 17). These subnuclei are reciprocally interconnected, allowing for ascending sensory flow and cross-modulation between noxious and innocuous signals (18–21). Given this ascending organization of trigeminal pathways, nociceptive and mechanosensory information from the orofacial region is transmitted to higher-order thalamic and cortical structures that integrate the sensory and affective dimensions of pain. Several other brain regions involved in pain modulation and emotional processing, including the anterior cingulate cortex, nucleus accumbens, thalamus, striatum, globus pallidus, and hippocampus (3, 22–24), have been implicated in pain and affective comorbidities of TN due to the structural and functional alterations. However, whether any of these regions serves as a critical node that links orofacial pain and pain-related negative emotions, and through which neural pathways such integration occurs, remains elusive.

The thalamic parafascicular nucleus (PF), a higher-order thalamic nucleus, is well recognized for its roles in arousal, attention, and locomotion (25–27). Unlike typical sensory relay thalamic nuclei that primarily transmit modality-specific information to the cortex, anatomical and transcriptomic studies have revealed that the PF contains distinct neuronal populations with topographically organized projections to the striatum (28). In humans, deep brain stimulation (DBS) targeting the centromedian-parafascicular (CM-PF) complex has shown therapeutic benefits for a range of neuropsychiatric and motor-related disorders, including Parkinson disease and Tourette syndrome (29, 30). Notably, case reports suggest that CM-PF DBS, particularly when combined with other targets, alleviates refractory neuropathic and central pain syndromes (31, 32). In rodents, PF has been implicated in the regulation of allodynia associated with depression but not in allodynia resulting from peripheral tissue injury (33, 34). Despite these clinical applications and experimental findings, direct evidence for PF involvement in orofacial pain and the possible underlying circuit-level mechanisms that contribute to the progression of TN remain lacking.

Here, by integrating clinical neuroimaging with mechanistic studies using a chronic constriction injury of the infraorbital nerve (CION) mouse model, we show that the PF functions as a pivotal node in TN. Structural MRI reveals PF atrophy in patients with TN. Using the CION mouse model, we identify 2 distinct PF neuronal ensembles orchestrated by a hierarchical trigeminal-thalamus circuitry: (*i*) a direct projection from GABAergic neurons in the oral part of spinal trigeminal nucleus (Sp5O) to the lateral PF (lPF) to modulate sensory pain and (*ii*) a trisynaptic glutamatergic pathway from the Sp5O, via the lateral parabrachial nucleus (lPBN), to the medial PF (mPF) that selectively encodes affective components. These 2 ensembles independently regulate the multidimensional symptoms of TN, with sex-specific differences emerging from the earlier hyperactivation of the anxiety-encoding ensemble in females. Critically, reciprocal excitatory projections between the mPF and lPBN establish a positive feedback loop, which drives pain chronicity. Molecular profiling identifies a *Col25a1*/*Syn2*-expressing mPF subpopulation, expressing genes encoding collagen type XXV alpha 1 chain (*Col25a1*) and synapsin II (*Syn2*), as the cellular substrate of this loop. Notably, disrupting this positive feedback loop attenuates both pain nociceptive and comorbid affective symptoms in the TN mouse model. Together, these findings reveal a circuit- and cell type–specific mechanism by which the PF orchestrates the multidimensional symptoms of TN and suggest a potential therapeutic avenue targeting chronic orofacial pain and its comorbidities.

## Results

### PF hyperactivity drives orofacial pain and comorbid anxiety.

**Figure 1:**
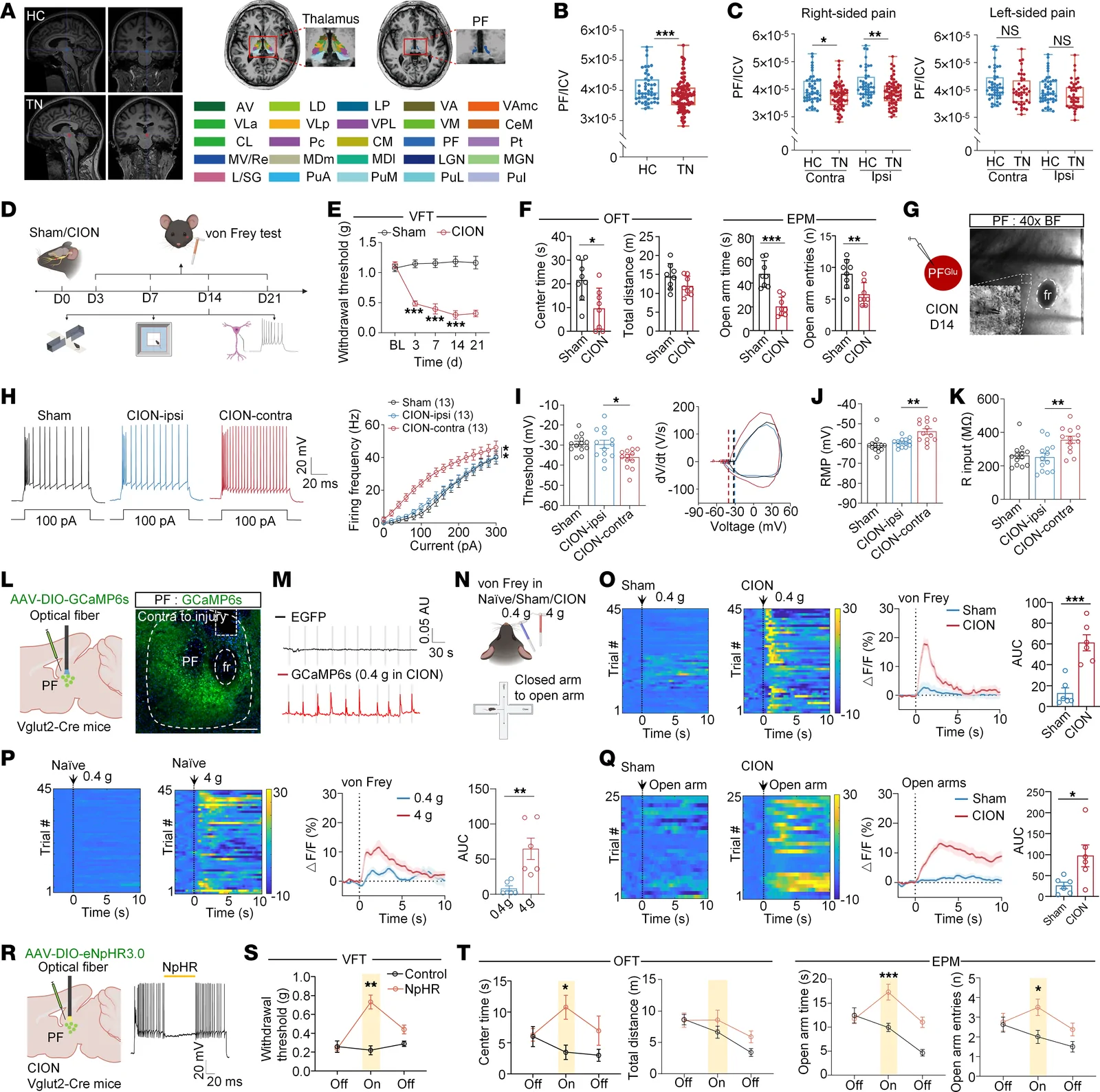
Hyperactivity of PF^Glu^ neurons mediates orofacial pain and comorbid anxiety of TN. (**A**) Representative fMRI T1 images of TN and HC. (**B**) Ratio of PF gray matter volume (GMV) to intracranial volume (48 HC and 111 TN patients). (**C**) Comparison of PF GMV of different pain sides. (**D**) Schematic of experiments. (**E**) Withdrawal threshold of sham and CION mice (*n* = 8 mice for each group). (**F**) Behavioral performance of sham and CION mice 2 weeks after surgery. (**G**) Location of the recorded neurons in PF slice. (**H**) Representative AP traces evoked by depolarizing current in PF^Glu^ neurons. (**I**–**K**) AP threshold, phase plots (**I**), RMP (**J**), and R input (**K**) in PF^Glu^ neurons. (**L**) Schematic of virus injection and example image of GCaMP6s (scale bar, 200 μm). (**M**) Representative traces of GCaMP6s signals in CION mice. (**N**) Experimental diagram for GCaMP6s recordings. (**O**–**Q**) Heatmaps, averaged normalized traces, and quantification (AUC in panels) of changes in GCaMP6s signals in sham/CION (**O**) and naive mice (**P**) receiving von Frey (0.4 g or 4 g) stimulation on the skin near the center of the vibrissa pad (V2 territory), and when sham/CION mice moved from closed arm to open arm in EPM (**Q**). (**R**) Schematic of virus injection and patch clamp recording in a PF slice. (**S** and **T**) Behavioral performance of CION mice in VFT (**S**), OFT, and EPM (**T**) with 3 min light-off, 3 min light-on, and 3 min light-off (*n* = 8 mice). Data are presented as means ± SEM. Unpaired Student’s *t* test for **B**, **F**, **O**–**Q**; 1-way ANOVA with Tukey’s post hoc test for **C** and **I**–**K**; and 2-way ANOVA with Tukey’s post hoc test for **E**, **H**, **S**, **T**; *P < 0.05, **P < 0.01, ***P < 0.001.

We analyzed a publicly available functional MRI (fMRI) dataset of patients with TN from OpenNeuro (dataset ds005713), in which the thalamus was segmented into 25 separate nuclei (Figure 1A). Gray matter volume in the PF contralateral to the pain side was significantly reduced in patients with TN compared with healthy controls (HCs) (Figure 1B and Supplemental Figure 1A; supplemental material available online with this article; https://doi.org/10.1172/JCI206862DS1). The PF volume was reduced bilaterally in patients with right-sided pain, though no generalized left-right asymmetry was observed (Figure 1C and Supplemental Figure 1B). PF atrophy did not correlate with pain severity, pain duration, or neurovascular compression status (Supplemental Figure 1, D and E). These findings suggest the PF as a potential thalamic nucleus implicated in the pathophysiology of TN.

To investigate the functional involvement of PF in the progression of TN, we generated the CION mouse model with injury to the right infraorbital nerve, which showed a marked reduction in right-sided orofacial pain thresholds beginning at 3 days postinjury and stabilizing by days 14 and 21 (Figure 1, D and E). These mechanical hypersensitivity symptoms were accompanied by increased nonevoked responses, such as prolonged facial grooming behavior, indicative of ongoing orofacial discomfort (Supplemental Figure 1F). On day 14, male CION mice exhibited anxiety-like behaviors, reflected by the decreased center time in the open field test (OFT) and reduced time and entries in the open arms of the elevated plus maze (EPM) (Figure 1F).

Next, we performed whole-cell patch-clamp recordings of PF^Glu^ neurons to assess the intrinsic excitability following CION-induced TN (Figure 1G). We focused on glutamatergic neurons because the PF is predominantly composed of excitatory neurons, with minimal inhibitory populations, making them the principal neuronal subtype involved in PF function and output (28). Whereas no change in firing rate was detected in the ipsilateral PF^Glu^ neurons, the frequencies of the current-evoked action potentials (APs) in the contralateral PF^Glu^ neurons were substantially increased 14 days after CION (Figure 1H). In addition, a more hyperpolarized AP threshold, a more depolarized resting membrane potential (RMP), and an increased input resistance (R input) were observed (Figure 1, I–K), suggesting elevated intrinsic excitability of PF^Glu^ neurons after CION. There were no differences in the amplitude, rise time, and width of the AP in PF^Glu^ neurons (Supplemental Figure 1G). We further recorded spontaneous excitatory (sEPSC) and inhibitory (sIPSC) postsynaptic currents in bilateral PF^Glu^ neurons. Following CION, there was a significant increase in the frequency of sEPSCs without a change in amplitude, accompanied by a marked reduction in both the frequency and amplitude of sIPSCs of the contralateral PF^Glu^ neurons (Supplemental Figure 1, H–M). These results reveal a presynaptic E/I imbalance of contralateral PF^Glu^ neurons, characterized by enhanced excitatory and diminished inhibitory inputs, indicative of maladaptive synaptic remodeling within PF-associated circuits following CION.

Based on the selective functional changes observed in the contralateral PF, we injected an adeno-associated virus (AAV) encoding Cre-dependent GCaMP6s into the contralateral PF of Vglut2-Cre mice to investigate activity changes of PF^Glu^ neurons associated with orofacial pain and anxiety-like behaviors, with behaviorally coupled calcium imaging performed 14 days after CION (Figure 1L). We detected a significant increase of GCaMP6 fluorescence following a weak stimulation (0.4 g von Frey filament) in CION mice (Figure 1, M–O), whereas in naive mice, only a suprathreshold stimulation (4 g von Frey filament) elicited a significant increase in GCaMP6 intensity in PF^Glu^ neurons (Figure 1P). Additionally, CION mice exhibited elevated neuronal activity during transitions from the closed to open arms in the EPM (Figure 1Q), and the activity showed a decreasing trend when mice exited the open arms (Supplemental Figure 1, N and O). These results indicate that PF^Glu^ neurons encode both orofacial pain and anxiety-like emotional states.

To determine if the hyperactivity of PF^Glu^ neurons is primarily involved in orofacial pain in TN or its anxiety-like comorbidity, or both, a Cre-dependent AAV encoding inhibitory opsin NpHR (halorhodopsin) was injected into the contralateral PF of Vglut2-Cre mice (Figure 1R). As expected, light activation of NpHR-mCherry effectively silenced neuronal firing, and it attenuated multiple pain- and affect-related behaviors in CION mice, leading to increased orofacial mechanical thresholds and reduced face-grooming duration, as well as decreased anxiety-like behaviors and increased conditioned preference for the stimulation-paired side (Figure 1, S and T, and Supplemental Figure 2, A and B). Consistently, chemogenetic inhibition of PF^Glu^ neurons via hM4Di produced similar analgesic and anxiolytic effects (Supplemental Figure 2, C–E). Conversely, chemogenetic activation of PF^Glu^ neurons under physiological conditions reduced orofacial mechanical thresholds and induced anxiety-like behaviors (Supplemental Figure 2, F–H). Together, these results demonstrate that heightened excitability of PF^Glu^ neurons is both necessary and sufficient to drive orofacial pain and comorbid anxiety following CION.

A previous study demonstrated that the CION model of TN induces central sensitization, as evidenced by tactile allodynia not only in the whisker pads but also in both hind paws (35). This finding suggests that CION-induced pain hypersensitivity extends beyond the trigeminal territory, implicating central pain-processing mechanisms. We therefore measured paw withdrawal thresholds in CION mice. On day 14 after CION surgery, paw withdrawal thresholds were significantly reduced compared with baseline values (Supplemental Figure 2I). Given the role of the PF in pain modulation, we next investigated whether PF activity contributes to this extratrigeminal hypersensitivity. Optogenetic inhibition of the PF in the CION model alleviated hind paw allodynia (Supplemental Figure 2J). These results suggest that PF activity is required for the hind paw tactile hypersensitivity observed after CION surgery.

### A direct Sp5O^GABA^-PF^Glu^ projection mediates orofacial pain.

**Figure 2:**
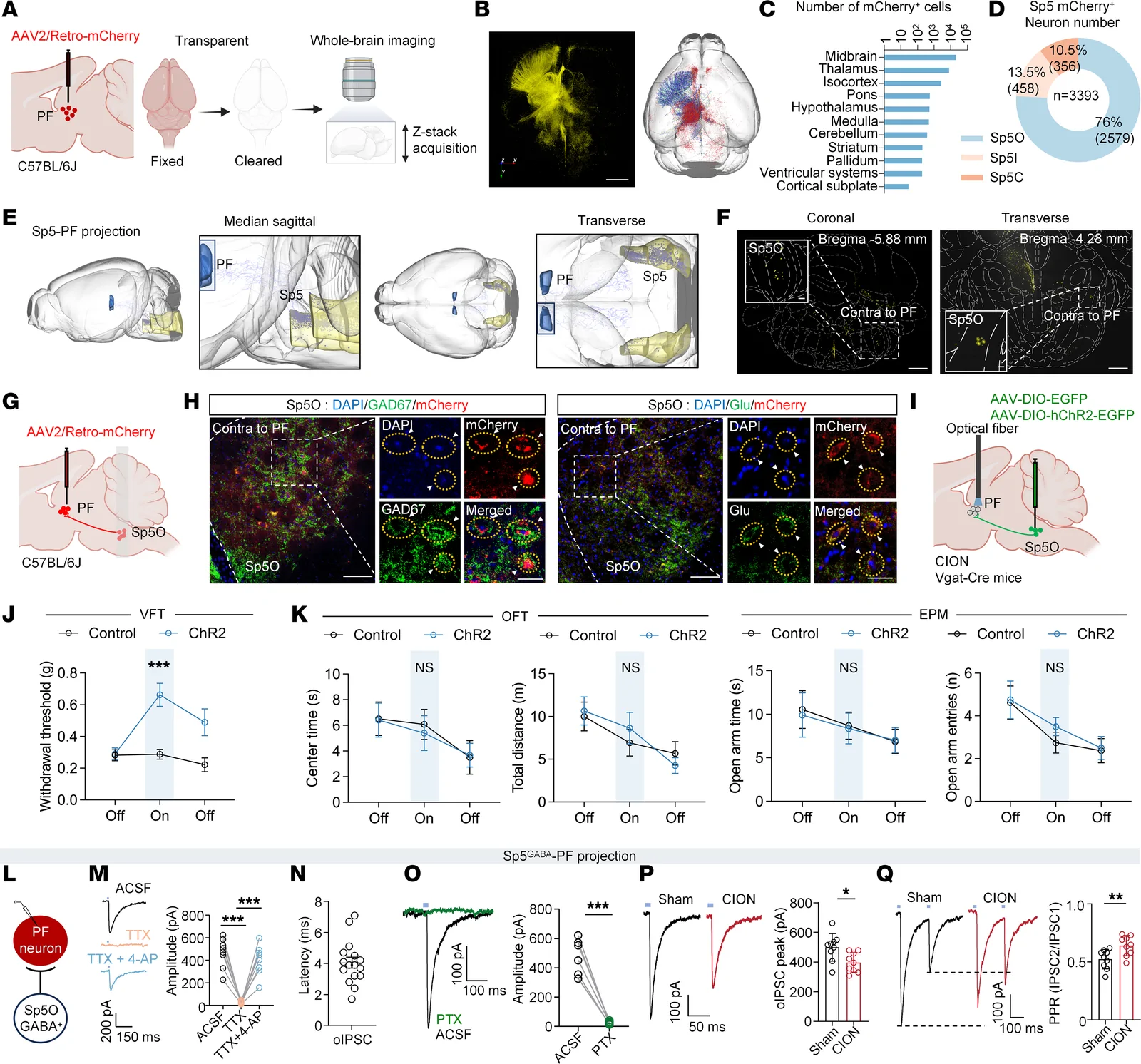
Projection of Sp5O^GABA^ neurons to the PF specifically mediates orofacial pain. (**A**) Schematic of virus injection and workflow of whole-brain transparency. (**B**) Fluorescence reconstruction image of a mouse brain in the transverse plane (scale bar, 200 mm) and 3-dimensional reconstruction images of somata (red) and fibers (blue). (**C** and **D**). Quantification of fluorescence-labeled cell somata in different upstream brain regions (**C**) and different subregions of the Sp5 (**D**). (**E**) Anatomical connectivity diagrams illustrating the Sp5-PF circuit. (**F**) Coronal and transverse sections of Sp5O, showing fluorescence-labeled cell somata (scale bar, 200 μm). (**G** and **H**) Schematic of virus injection (**G**), immunostaining images of GAD67 and Glu (**H**). (**I**) Schematic of virus injection. (**J** and **K**) Behavioral performance of CION mice in the von Frey test (**J**), OFT, and EPM (**K**) with 3 min light-off, 3 min light-on, and 3 min light-off (*n* = 8 mice for each group). (**L**) Schematic of patch clamp on PF neurons that receive Sp5O^GABA^ projection. (**M**) Optogenetic activation of Sp5O^GABA^ input-evoked oIPSCs in PF neurons, their complete inhibition by application of TTX, and restoration by 4-AP. (**N**) oIPSC latency upon light stimulation (*n* = 16 cells from 3 mice). (**O**) Inhibition of oIPSCs by PTX and quantification of oIPSC peak amplitude. (**P**) Example traces and peak amplitude of blue light–evoked (5 ms, 2 mW) oIPSCs in PF neurons from sham and CION mice. (**Q**) Example traces and PPR of blue light–evoked (5 ms, 2 mW, 200 ms interval) oIPSCs recorded in PF neurons from sham and CION mice. Data are presented as means ± SEM. Paired Student’s *t* test for **O**, unpaired Student’s *t* test for **P** and **Q**, 1-way ANOVA with Tukey’s post hoc test for **M**, and 2-way ANOVA with Tukey’s post hoc test for **J** and **K**; *P < 0.05, **P < 0.01, ***P < 0.001.

We sought to define the circuit-based mechanisms by which PF^Glu^ neurons control orofacial pain and anxiety. To characterize the projection profile that innervates PF, we injected mCherry-expressing retrograde virus into the PF and visualized retrograde labeling with whole-brain tissue clearing (Figure 2A). A total of 11 brain regions showed direct projections to PF, as shown by the dense mCherry labeling of upstream neurons (Figure 2, B and C, and Supplemental Figure 3A). We observed that the Sp5 sends direct projections to the PF, with approximately 76% of PF-projecting neurons arising from Sp5O (Figure 2, D and E). Moreover, in both coronal and transverse sections, retrogradely labeled neurons were found predominantly concentrated in the Sp5O contralateral to the PF injection site, with only rare instances found in the ipsilateral Sp5O (Figure 2F). We immunostained glutamate decarboxylase 67 (GAD67, a GABA synthesizing enzyme) and glutamate (Glu) to determine the neurotransmitter identity of mCherry-labeled neurons projecting from Sp5O to the PF, revealing that approximately 67.3% of these neurons expressed GAD67, while approximately 30.3% expressed Glu (Figure 2, G and H, and Supplemental Figure 5A).

To ascertain the roles of the Sp5O^GABA^-PF and Sp5O^Glu^-PF circuits in orofacial pain and anxiety, we injected Cre-dependent AAVs encoding the excitatory opsin channelrhodopsin-2 (ChR2) and the inhibitory NpHR into the Sp5O of Vgat-Cre and Vglut2-Cre mice, respectively (Figure 2I and Supplemental Figure 4C), and implanted optical fibers in the PF contralateral to the Sp5O. Optogenetic activation of Sp5O^GABA^-PF terminals selectively increased orofacial pain thresholds and reduced face-grooming duration in CION mice without altering anxiety-like behaviors and was associated with a trend toward increased preference in the conditioned place preference (CPP) assay (Figure 2, J and K, and Supplemental Figure 4, A and B). However, optogenetic inhibition of Sp5O^Glu^-PF neuron terminals produced no detectable changes in either pain thresholds or anxiety (Supplemental Figure 4, D and E). These results indicate that the Sp5O^GABA^-PF pathway mediates CION-induced hyperalgesia.

With the same virus strategy using Cre-dependent ChR2 to label Sp5O^GABA^ neurons, we recorded optically evoked inhibitory postsynaptic currents (oIPSCs) in the PF neurons upon blue light activation of Sp5O terminals in brain slices (Figure 2L). The oIPSCs were abolished after the application of the sodium channel blocker tetrodotoxin (TTX), which blocks APs in all neurons, but restored by the coapplication of 4-aminopyride (4-AP) — a potassium channel antagonist that enables ChR2-mediated depolarization of local presynaptic terminals (Figure 2M). Synchronized oIPSCs were observed after light activation, with a rapid onset characterized by a mean latency of 4.05 ms (Figure 2N), indicating that Sp5O^GABA^ neurons directly inhibit PF^Glu^ neurons. Application of the GABA_A_ receptor blocker picrotoxin (PTX) abolished these currents, verifying their GABAergic origin (Figure 2O). The peak amplitude of oIPSCs was significantly reduced in CION mice compared with Sham controls, indicating a diminished inhibitory Sp5O-PF input (Figure 2P). To investigate whether the attenuation of Sp5O^GABA^-PF inhibitory input in TN involves pre- or postsynaptic mechanisms, we measured the paired pulse ratio (PPR) of IPSCs evoked by paired blue light pulses (5 ms, 2 mW, 100 ms interval). The PPR was significantly higher in PF neurons of CION mice than sham controls (Figure 2Q), indicating that CION impairs Sp5O^GABA^-PF transmission, at least in part, via a presynaptic mechanism. Thus, hyperactivity of PF^Glu^ neurons in CION mice likely arises from presynaptic disinhibition due to diminished inhibitory input from Sp5O^GABA^ neurons.

Analysis of the Allen Brain Cell Atlas revealed abundant parvalbumin (PV) neurons in Sp5O, with sparse somatostatin (SST) and vasoactive intestinal peptide neurons; notably, PV neurons showed substantial overlap with GABAergic neurons (Supplemental Figure 5B). To specifically target this subset and verify their projection to the PF, PV-Cre mice were injected with AAV2/retro-hSyn-Flp-mCherry into the PF and AAV-hSyn-Con Fon-EYFP into the Sp5O contralateral to the PF (Supplemental Figure 5, C and D). Approximately 62.7% of mCherry-labeled neurons in Sp5O coexpressed EYFP (Supplemental Figure 5E), mirroring the fraction of GABAergic neurons and confirming the PV^+^ neurons constituting the predominant inhibitory projections to the PF. To determine whether this specific molecular subtype drives the observed sensory modulation, we performed optogenetic manipulations of the Sp5O^PV^-PF circuit using a strategy analogous to our Vgat-Cre experiments (Supplemental Figure 5F). Consistent with the Vgat-based results, optogenetic activation of Sp5O^PV^ terminals in the PF significantly elevated orofacial pain thresholds in CION mice without altering anxiety-like behaviors (Supplemental Figure 5, G and H). These findings identify inhibitory neurons among the PV-expressing population in Sp5O that selectively gates the sensory, but not the affective, dimension of TN.

### Enhanced Sp5O^Glu^-lPBN^Glu^-PF^Glu^ transmission encodes pain-related anxiety.

**Figure 3:**
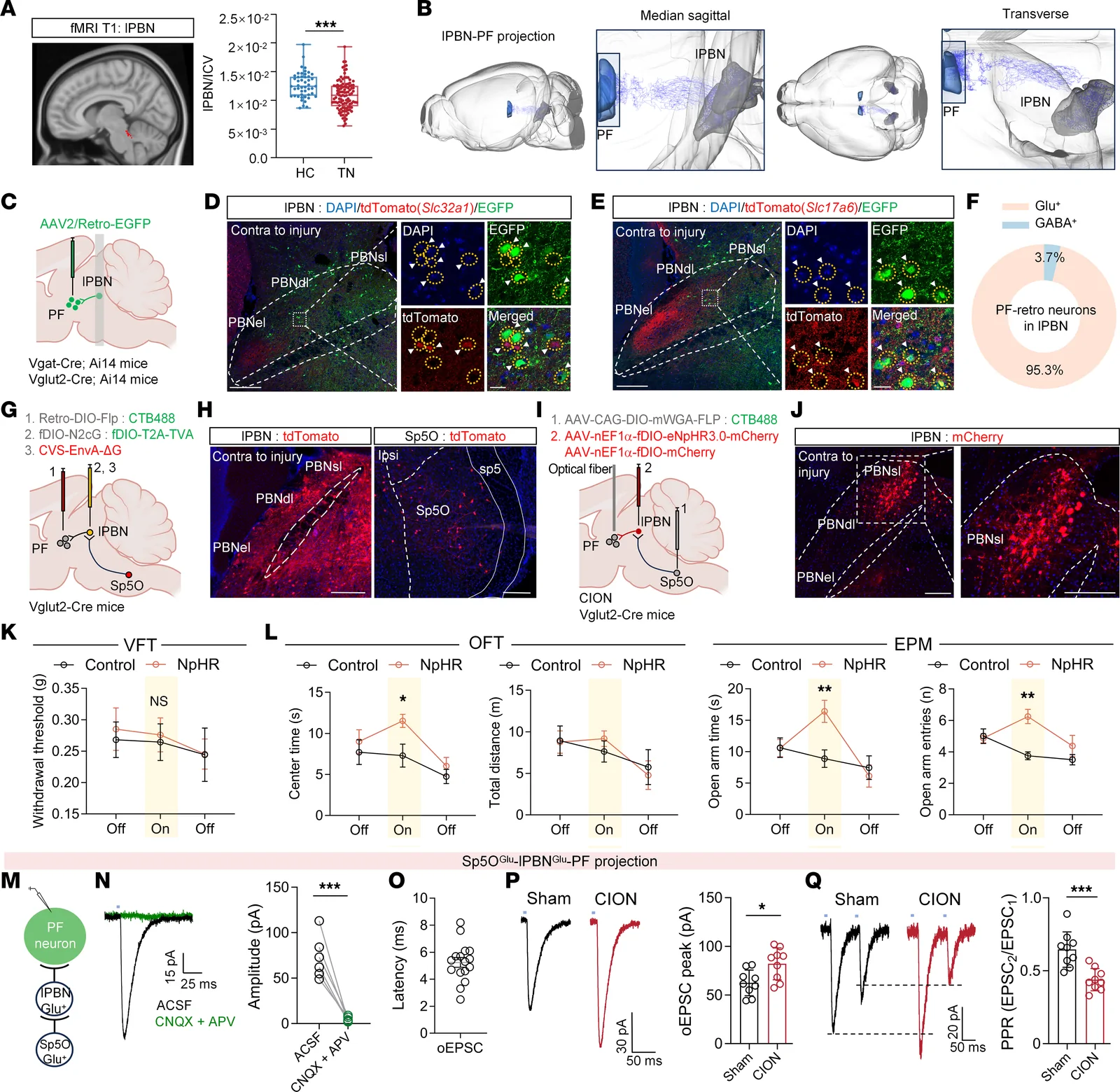
Excitatory projections in the Sp5O^Glu^-lPBN^Glu^-PF^Glu^ pathway specifically mediate pain-related anxiety. (**A**) A representative lPBN segmentation from 1 individual, with the lPBN highlighted (*left*), and the ratio of lPBN GMV to intracranial volume in HC and TN (*right*). (**B**) Anatomical connectivity diagrams illustrating the lPBN-PF circuit. (**C**–**F**) Schematic of virus injection (C), tdTomato^+^ GABAergic neurons (**D**) and tdTomato^+^ glutamatergic neurons (**E**) (scale bar, 50 μm; zoomed image, 20 μm), and statistics on the proportion of GABAergic and glutamatergic neurons in lPBN that project to PF (**F**). (**G** and **H**) Schematic of virus injection (**G**), representative images of tdTomato expression in lPBN and Sp5O (**H**) (scale bar, 50 μm). (**I**–**L**) Schematic of virus injection (**I**), representative images of mCherry expression in lPBN (**J**) (scale bar, 50 μm), and behavioral performance of CION mice in the von Frey test (**K**), OFT, and EPM (**L**) with 3 min light-off, 3 min light-on, and 3 min light-off (*n* = 8 mice for each group). (**M**) Schematic of patch clamp on PF neurons that receive Sp5O^Glu^-lPBN^Glu^ projection. (**N**) Optogenetic stimulation of lPBN^Glu^ input–evoked oEPSCs in PF neurons and their inhibition by coapplication of CNQX and APV. (**O**) oEPSC latency upon light stimulation (*n* = 16 cells from 3 mice). (**P**) Example traces and peak amplitude of blue light–evoked (5 ms, 2 mW) oEPSCs in PF neurons from sham and CION mice. (**Q**) Example traces and PPR of blue light–evoked (5 ms, 2 mW, 100 ms interval)oEPSCs recorded in PF neurons from sham and CION mice. Data are presented as means ± SEM. Paired Student’s *t* test for **N**, unpaired Student’s *t* test for **P** and **Q**, and 2-way ANOVA with Tukey’s post hoc test for **K** and **L**; *P < 0.05, **P < 0.01, ***P < 0.001.

Considering the selective role of the Sp5O^GABA^-PF^Glu^ pathway in modulating pain sensation in TN and the observation that manipulating PF^Glu^ neurons affected both orofacial pain and comorbid anxiety, we hypothesized that a distinct upstream projection to the PF is likely responsible for encoding pain-related anxiety. Among the brain regions identified in our connectome analysis, the lPBN in the pons emerged as the predominant input source to the PF (Supplemental Figure 6A), consistent with previous anterograde tracing studies using various Cre-driver lines to label PBN efferents (36, 37). Using the same fMRI dataset from the TN patient cohort described above, we further performed anatomical segmentation of the lPBN and quantified its gray matter volume contralateral to the pain side. Patients with TN showed a significant reduction in lPBN gray matter volumes compared with HCs (Figure 3A). To explore the potential cellular substrates underlying this structural alteration, we examined the anatomical organization of the lPBN/PF pathway in mice. Using the same retrograde viral tracing and whole-brain clearing strategy described above, we observed dense fiber projections from the lPBN to the PF, along with numerous mCherry-positive somata within the lPBN (Figure 3B and Supplemental Figure 6B). To characterize the neurotransmitter identity of the PF-projecting lPBN neurons, a retrograde virus tracer was injected into the PF of Vglut2-Cre Ai14 or Vgat-Cre Ai14 mice (Figure 3C). The vast majority (~95%) of the retrogradely labeled cells colocalized with Vglut2^+^ neurons, whereas only a small fraction colocalized with Vgat^+^ neurons (Figure 3, D–F). To identify the upstream inputs to PF-projecting lPBN^Glu^ neurons, we first injected a Cre-dependent Flp-expressing retrograde tracing virus into the PF of Vglut2-Cre mice and an Flp-dependent retrograde trans-monosynaptic tracing system into the lPBN (Figure 3G and Supplemental Figure 6C). Three weeks later, Cvs-EnvA-G-tdTomato was injected into the lPBN to allow for monosynaptic retrograde labeling of upstream inputs to PF-projecting lPBN^Glu^ neurons. We found a robust tdTomato expression in bilateral Sp5O (Figure 3H and Supplemental Figure 6, D–F), indicating that lPBN neurons projecting to the PF receive direct synaptic input from the Sp5O. In addition, tdTomato-labeled neurons were also observed in several other brain regions, including cortex, pallidum, striatum, midbrain, and hypothalamus, as well as other areas (Supplemental Figure 6, G and H).

To confirm the functional connections and essential roles of Sp5O^Glu^-lPBN^Glu^-PF^Glu^ in vivo, we employed a combinatorial strategy that permits direct modulation of PF-projecting lPBN^Glu^ neurons that are also innervated by Sp5O^Glu^ neurons. A Cre-dependent modified wheat germ agglutinin–Flp (mWGA-Flp) was injected into the Sp5O ipsilateral to CION injury of Vglut2-Cre mice, enabling Sp5O^Glu^ neurons to express mWGA-Flp and trans-synaptically deliver Flp recombinase to their postsynaptic targets in the lPBN (38). Concurrently, an Flp-dependent NpHR was injected into the lPBN contralateral to CION injury, allowing selective expression of the inhibitory opsin NpHR in lPBN neurons innervated by Sp5O^Glu^ neurons (Figure 3I and Supplemental Figure 6I). mCherry-positive neurons were observed in the lPBN and were primarily localized to superior-lateral PBN (PBNsl) (Figure 3J). Optogenetic inhibition of the Sp5O^Glu^-lPBN^Glu^-PF^Glu^ pathway selectively alleviated anxiety-like behaviors and produced a significant conditioned preference for the stimulation-paired chamber without affecting orofacial pain and face-grooming behavior in CION mice (Figure 3, K and L, and Supplemental Figure 6, J and K). These results not only confirm the excitatory effect of Sp5O^Glu^-lPBN input on PF-projecting lPBN neurons in vivo but also demonstrate that this pathway selectively contributes to pain-related anxiety.

With a similar virus strategy injecting the Cre-dependent mWGA-Flp into the Sp5O and Flp-dependent ChR2 into the lPBN, optically evoked excitatory postsynaptic currents (oEPSCs) in PF neurons were recorded in brain slices upon blue light stimulation (Figure 3M). These oEPSCs were completely abolished by coapplication of the AMPA receptor antagonist 6-cyano-7-nitroquinoxaline-2,3-dione (CNQX) and NMDA receptor antagonist D-2-amino-5-phosphonovaleric acid (APV) (Figure 3N), confirming the excitatory nature of this projection. We also found synchronized oEPSCs after the light stimulation, with a rapid onset characterized by a mean latency of 5.24 ms (Figure 3O). The peak amplitude of oEPSCs was significantly increased in CION mice compared with sham controls (Figure 3P), indicating an enhancement of the Sp5O-lPBN-PF projection. Furthermore, PPR of EPSCs evoked by paired blue light pulses (5 ms, 2 mW, 100 ms interval) was significantly decreased in PF neurons of CION mice (Figure 3Q), suggesting a presynaptic facilitation of the Sp5O^Glu^-lPBN^Glu^-PF^Glu^ pathway. Collectively, these results highlight the critical role of the Sp5O-lPBN-PF circuit in mediating the affective comorbidities following CION.

Although previous studies have reported that nociceptive inputs from the infraorbital branch (V2) primarily target the Sp5C subregion (39, 40), we further investigated the structural and functional connectivity of the Sp5C-Sp5O pathway. A retrograde Cre-dependent AAV was injected into the Sp5O (Supplemental Figure 7, A and B), and retrograde tracing confirmed a direct glutamatergic projection from Sp5C to Sp5O (Supplemental Figure 7C). To assess whether this pathway is altered following CION, we expressed Cre-dependent ChrimsonR in Sp5C neurons and Cre-dependent GCaMP6s in Sp5O neurons (Supplemental Figure 7, D–F). By combining optogenetic stimulation of Sp5C terminals with fiber photometry recording within the Sp5O, we observed that light-evoked calcium signals were significantly enhanced in CION mice compared with the sham group (Supplemental Figure 7, G–I). These results indicated that the functional connectivity of the Sp5C-Sp5O projection is potentiated following CION. Together, these results delineate the upstream brainstem circuit framework linking peripheral trigeminal input to ascending pathways and reinforce the critical role of Sp5O in this process.

### Segregated input-defined neuronal ensembles in the PF exhibit sequential activation and distinct roles in TN.

**Figure 4:**
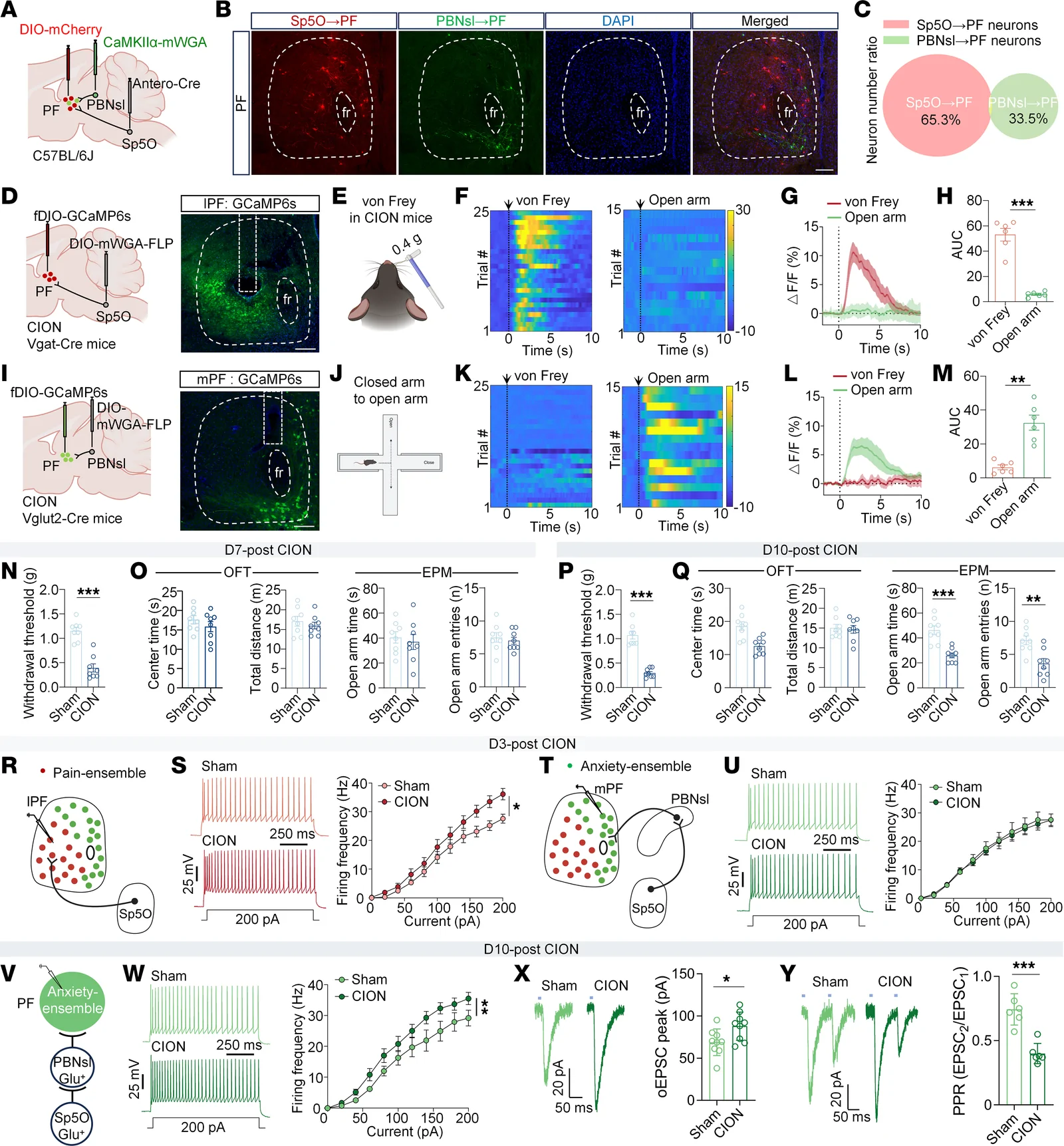
Presence of segregated PF neuronal ensembles with distinct spatiotemporal activation. (**A**–**C**) Schematic of virus injection (**A**), representative images of mCherry/EGFP expression (scale bar, 200 μm) (**B**), and statistics for the proportion of PF neurons receiving Sp5O or PBNsl projection (**C**). (**D**–**M**) Schematic and representative images of GCaMP6s (**D** and **I**) expression (scale bar, 200 μm), and experimental diagrams of GCaMP6s recordings (**E** and **J**), as well as heatmaps (**F** and **K**), averaged normalized traces (**G** and **L**), and AUC statistics (**H** and **M**) of GCaMP6s signals (**F**–**H**) or moving from closed to open arms in EPM (**K**–**M**). (**N** and **O**) Behavioral performance of mice in the von Frey test (**N**), OFT, and EPM (**O**) on day 7 after surgery. (**P** and **Q**) Behavioral performance of male mice in the von Frey test (**P**), OFT, and EPM (**Q**) on day 10 after surgery. (**R**–**U**) Schematic of patch clamp on PF neurons that receive Sp5O (**R**) or PBNsl (**T**) projection and examples of AP traces in PF pain-ensemble (**S**) or anxiety-ensemble (**U**) neurons on day 3 after surgery (*n* = 12 cells from 3 mice). (**V**) Schematic of patch clamp on PF neurons. (**W**) Examples of AP traces in PF anxiety-ensemble neurons from sham and CION mice on day 10 after surgery (*n* = 10 cells from 3 mice). (**X**) Example traces and peak amplitude of blue light–evoked (5 ms, 2 mW) oEPSCs in PF anxiety-ensemble neurons on day 10 after surgery. (**Y**) Example traces and PPR of blue light–evoked (5 ms, 2 mW, 100 ms interval) oEPSCs recorded in PF anxiety-ensemble neurons on day 10 after surgery. Data are presented as means ± SEM. Unpaired Student’s *t* test for **H**, **M**–**Q**, **X**, and **Y** and 2-way ANOVA with Tukey’s post hoc test for **S**, **U**, and **W**; *P < 0.05, **P < 0.01, ***P < 0.001.

Integrating the connectivity and functional organization of the 2 PF-projecting circuits, our data strongly suggest that each pathway selectively recruits distinct neuronal ensembles within the PF. To test this hypothesis, we injected CaMKIIa-dependent mWGA-EGFP into the PBNsl ipsilateral to PF (Supplemental Figure 8A), an anterograde trans-synaptic Cre virus into the Sp5O contralateral to PF, and a Cre-dependent mCherry-expressing virus into the PF of wild-type C57BL/6J mice (Figure 4A). Among the labeled PF neurons, approximately 33.5% expressed only EGFP, while approximately 65.3% were labeled with anterograde virus-dependent mCherry, with no colocalization observed between EGFP and mCherry. This suggests that PBNsl and Sp5O innervate 2 largely distinct populations of PF neurons (Figure 4, B and C). Notably, the ensemble receiving PBNsl input was anatomically confined to the mPF.

To delineate the response patterns of the 2 neuronal ensembles, we performed real-time calcium imaging in circuit-specific PF^Glu^ neurons. Cre-dependent mWGA-Flp was injected into either the Sp5O of Vgat-Cre mice or the PBNsl of Vglut2-Cre mice, while Flp-dependent GCaMP6s was injected into the PF to direct selective GCaMP6s expression in the PF^Glu^ neurons innervated by Sp5O^GABA^ or PBNsl^Glu^, respectively. Neurons innervated by Sp5O^GABA^ were predominantly localized to the lPF, whereas those innervated by PBNsl^Glu^ were confined to the mPF (Figure 4, D and I). Calcium imaging revealed that PF neurons receiving input from Sp5O^GABA^ neurons exhibited robust responses to orofacial pain stimulation but not emotionally salient stimuli (Figure 4, E–H). Conversely, PF neurons receiving input from PBNsl^Glu^ neurons were strongly activated during transitions from the closed to open arms in the EPM but remained unresponsive to orofacial stimuli (Figure 4, J–M). Taken together, orofacial pain and anxiety stimulation selectively recruit distinct neuronal ensembles within the PF, suggesting that the inhibitory Sp5O and excitatory PBNsl inputs differentially shape PF’s functional roles in pain and emotional processing.

Consistent with clinical observations that mood disorders often emerge later than the onset of pain sensitizations, male CION mice developed orofacial pain as early as day 3 post-CION (Figure 1E), whereas anxiety-like behaviors were undetectable on day 7, but became consistently observed around day 10 (41–45) (Figure 4, N–Q). We next asked whether the activation of the 2 input-defined PF neuronal ensembles associated with pain and anxiety, respectively, also exhibits a temporal sequence. To address this, we performed in vitro electrophysiological recordings on multiple days after CION. On day 3 post-CION in male mice, the PF neuronal ensemble receiving input from the Sp5O (hereafter referred to as the pain-ensemble) showed a marked increase in AP firing frequency (Figure 4, R and S), whereas the PBNsl-innervated ensemble (referred to as the anxiety-ensemble) showed no detectable changes in excitability (Figure 4, T and U). By day 10 post-CION, however, the PF anxiety-ensemble displayed a marked increase in excitability (Figure 4, V and W), along with increased oEPSCs (Figure 4X) and decreased PPR, specifically within the Sp5O^Glu^-lPBN^Glu^-PF^Glu^ circuit (Figure 4Y). These findings reveal a temporal activation of the 2 PF ensembles following CION, with delayed engagement of the anxiety-ensemble temporally aligned with the emergence of anxiety-like behaviors.

Given the higher clinical prevalence of TN in female patients, we also conducted experiments using female mice. Female CION mice developed robust unilateral orofacial mechanical hypersensitivity, characterized by a marked reduction in right-sided facial pain thresholds beginning at 3 days postinjury and reaching a stable level by days 14 and 21 (Supplemental Figure 8F). On day 14, female CION mice also exhibited anxiety-like behaviors, as reflected by decreased center time in the OFT and reduced time and entries in the open arms of the EPM (Supplemental Figure 8, G and H). Notably, whereas anxiety-like behaviors in male mice emerged around day 10 post-CION and remained undetectable at day 7 (Figure 4O), female mice displayed marked anxiety-like behaviors as early as day 7 post-CION (Supplemental Figure 8, I and J). We therefore next examined an earlier time point (day 3 post-CION) and found that anxiety-like phenotypes were not yet detectable at this stage in female mice (Supplemental Figure 8, K and L). These findings indicate that female mice develop TN-related sensory and affective phenotypes comparable to those observed in males but with a temporally earlier onset of anxiety-like behaviors.

To further determine whether this earlier behavioral transition in female mice was accompanied by altered recruitment of the PF anxiety-ensemble, we performed in vitro electrophysiological recordings at day 7 post-CION. PF neurons receiving input from the PBNsl exhibited a marked increase in AP firing frequency, together with a more hyperpolarized AP threshold, a more depolarized RMP, and increased input resistance (Supplemental Figure 8, M–P), indicating enhanced intrinsic excitability following CION. No significant differences were observed in AP amplitude, rise time, or width (Supplemental Figure 8, Q–S). Furthermore, the PF anxiety-ensemble within the Sp5O^Glu^-lPBN^Glu^-PF^Glu^ circuit exhibited increased oEPSC amplitudes and decreased PPR (Supplemental Figure 8, T–V), suggesting enhanced excitatory synaptic transmission within this pathway. Together, these findings indicate that the major TN-related sensory and affective phenotypes are largely comparable between sexes, while female mice exhibit earlier recruitment of the PF anxiety-associated circuit in parallel with the earlier emergence of anxiety-like behaviors.

### An lPBN-mPF-lPBN positive feedback loop drives orofacial pain chronicity.

**Figure 5:**
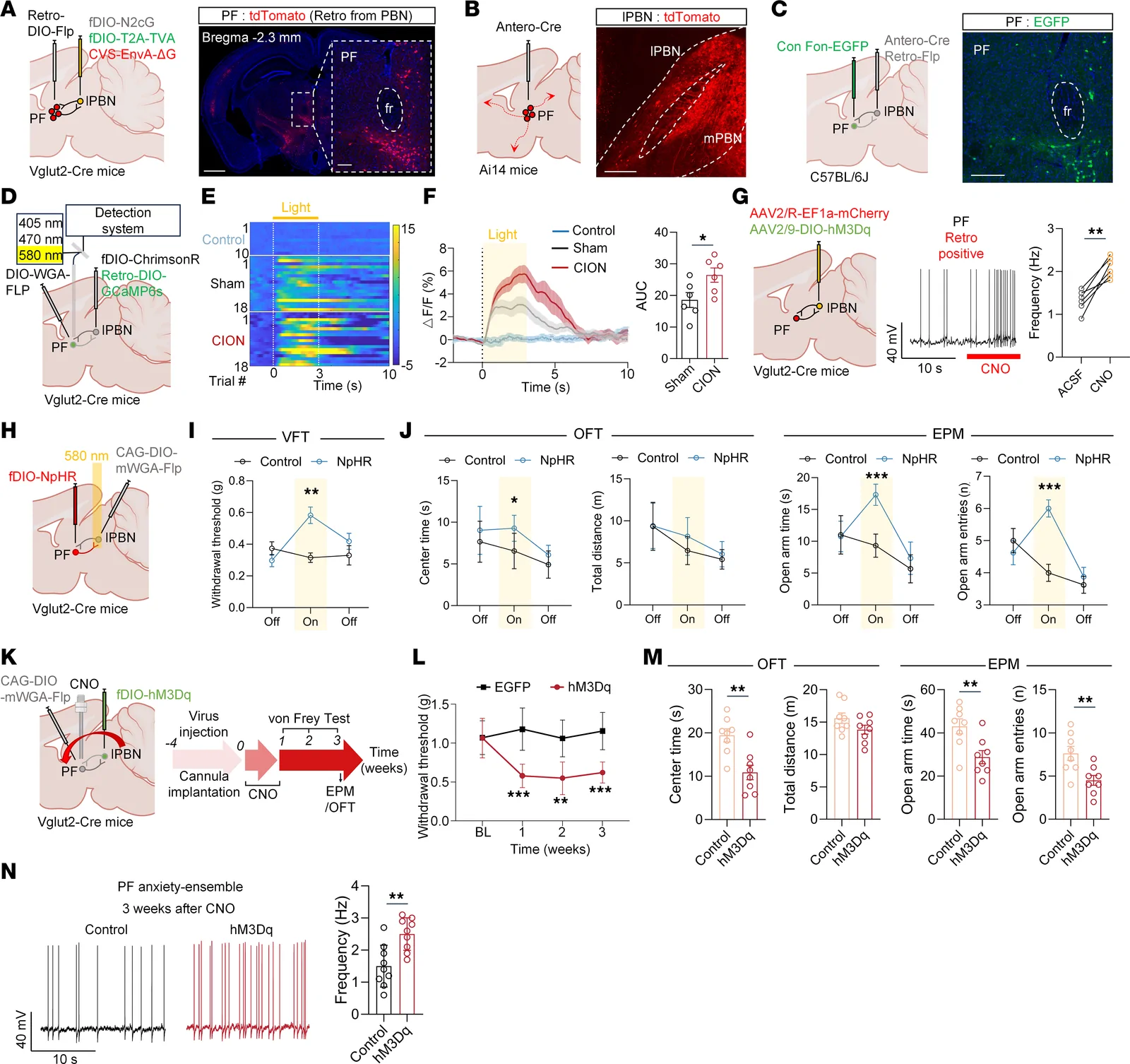
lPBN-mPF-lPBN forms a positive feedback loop that promotes pain chronicity. (**A**) Schematic of virus injection and representative images of tdTomato expression (scale bar, 1,000 μm; zoomed image, 100 μm). (**B**) Schematic of virus injection and tdTomato expression (scale bar, 200 μm). (**C**) Schematic of virus injection and EGFP expression in PF (scale bar, 200 μm). (**D**–**F**) Schematic (**D**), heatmaps (**E**), and averaged normalized traces and quantification (AUC) of GCaMP6s signals (**F**), showing the effect of optogenetic activating lPBN terminals on PF^Glu^ neurons that receive lPBN projections (control, *n* = 10 trials from 3 mice; sham/CION, *n* = 18 trials from 6 mice). (**G**) Schematic, representative AP recordings, and statistics showing the effect of chemogenetic activation of lPBN terminals on PF^Glu^ neurons that receive lPBN projections (*n* = 6 cells from 3 mice). (**H**–**J**) Schematic of virus injection (**H**), behavioral performance of CION mice in VFT (**I**), OFT, and EPM (**J**) with 3 min light-off, 3 min light-on, and 3 min light-off. (*n* = 8 mice.) (**K**) Schematic of injection of Cre-dependent mWGA-Flp virus into the PF and Flp-dependent chemogenetic activation virus into the lPBN, followed by CNO local administration in the PF of Vglut2-Cre mice. (**L** and **M**) Statistics of withdrawal threshold from VFT (**L**) and behaviors in the OFT and EPM (**M**) in the chemogenetic excitatory group and control mice during and/or after daily CNO administration for 1 week. (**N**) Spontaneous APs of PF anxiety-ensemble neurons of mice 3 weeks after CNO application (*n* = 9 cells from 3 mice for each group). Data are presented as means ± SEM. Paired Student’s *t* test for **G**, unpaired Student’s *t* test for **F**, **M**, and **N**, 2-way ANOVA with Tukey’s post hoc test for **I**, **J**, and **L**; *P < 0.05, **P < 0.01, ***P < 0.001.

We next investigated what upstream mechanisms might sustain the lPBN/PF circuit in the chronic state. Whole-brain analysis of inputs to lPBN^Glu^ neurons that project to the PF using the TVA-based retrograde tracing strategy revealed a substantial population of tdTomato-expressing neurons distributed specifically within the mPF (Figure 5A). This finding indicates that PF-projecting lPBN neurons receive direct feedback selectively from the mPF, but not the lPF, thereby forming an lPBN-mPF-lPBN feedback loop. To confirm the synaptic connection from the PF to the lPBN, a Cre-expressing anterograde virus was injected into the PF of Ai14 mice, resulting in robust tdTomato expression in both the lateral and medial PBN (Figure 5B).

To further validate that the lPBN-mPF-lPBN loop may constitute a closed, cell-to-cell reciprocal circuit, we employed a dual-recombinase strategy enabling selective labeling of mPF neurons that both receive projections from the lPBN and project back to it. A Cre-expressing anterograde virus and an Flp-expressing retrograde virus were coinjected into the lPBN, while a Cre- and Flp-dependent EGFP was injected into the PF. We observed that EGFP-expressing neurons were specifically localized to the mPF (Figure 5C), providing direct evidence that the lPBN-mPF-lPBN circuit forms a topographically organized, closed-loop circuit within the mPF.

To test if the lPBN^Glu^-mPF^Glu^-lPBN^Glu^ circuit is functional, we injected a Cre-dependent mWGA-Flp virus into the PF of Vglut2-Cre mice to enable trans-synaptic delivery of Flp to lPBN neurons receiving input from PF^Glu^ neurons. Simultaneously, an Flp-dependent ChrimsonR-expressing virus was injected into the lPBN, leading to the selective expression of ChrimsonR in lPBN neurons that are innervated by PF^Glu^ neurons. In addition, a retrograde Cre-dependent GCaMP6s-expressing virus was injected into the lPBN to label PF^Glu^ neurons projecting to the lPBN (Figure 5D). Optogenetics-coupled fiber photometry recordings revealed that optogenetic activation of terminals of lPBN neurons receiving PF input induced robust calcium signals in lPBN-projecting PF^Glu^ neurons, and this response was significantly enhanced in mice with CION (Figure 5, E and F), validating the increased excitatory effect of lPBN^Glu^ terminals on lPBN-projecting PF^Glu^ neurons post-CION. To verify the functional connection of this positive feedback loop, we coinjected a Cre-dependent hM3Dq virus and a retrograde mCherry-expressing virus into the lPBN of Vglut2-Cre mice (Figure 5G). Chemogenetic activation of lPBN^Glu^ terminals in the PF, via clozapine *N*-oxide (CNO) application in acute PF slices, significantly increased the firing rate of lPBN-projecting PF^Glu^ neurons, confirming a functional excitatory influence within the loop.

Since pain-induced anxiety in TN poses a substantial clinical challenge, and our data point to mPF activity as a key driver of pain persistence, we tested whether inhibiting the loop could reverse symptoms. Cre-dependent mWGA-Flp virus was injected into the lPBN of Vglut2-Cre mice, and an Flp-dependent NpHR was injected into the mPF (Figure 5H). Optogenetic inhibition of the PF terminals in lPBN substantially alleviated both orofacial pain and anxiety-like behaviors in CION mice (Figure 5, I and J, and Supplemental Figure 10A).

We then asked whether long-term activation of the mPF anxiety-ensemble would be sufficient to induce TN-like behaviors in naive mice. The Cre-dependent mWGA-Flp virus was injected into the mPF of Vglut2-Cre mice, with a cannula implanted into the mPF for CNO local application without affecting somatic activity in the lPBN; meanwhile, the Flp-dependent hM3Dq-expressing virus was injected into the lPBN (Figure 5K). Daily CNO administration for 1 week led to both anxiety-like behavior and a marked reduction in orofacial pain threshold. Notably, these effects persisted for over 2 weeks after CNO withdrawal, indicating a long-lasting impact of mPF anxiety-ensemble activation (Figure 5, L and M). Consistently, the elevated spontaneous firing frequency of the mPF anxiety-ensemble remained detectable even 3 weeks after CNO withdrawal (Figure 5N). These findings suggest that tuning up the lPBN-mPF-lPBN positive feedback loop by activating the PF anxiety-ensemble is sufficient to drive the long-term persistence of TN, establishing the critical role of this loop in pain chronicity.

To further examine the functional specificity of this effect within the PF, we next chronically activated lPF neurons by expressing hM3Dq and delivering CNO continuously for 1 week via cannula infusion (Supplemental Figure 9A). On the final day of CNO administration (week 1), mice exhibited decreased facial pain thresholds. However, this effect was not sustained after drug withdrawal, as pain thresholds returned to baseline levels during weeks 2 and 3 (Supplemental Figure 9B). In contrast with the persistent pain sensitization induced by chronic activation of the mPF anxiety-ensemble, chronic lPF activation failed to produce sustained posttreatment pain hypersensitivity. These findings further support the functional specificity of the mPF-centered circuit in driving long-term pain chronicity.

Clinically, selective serotonin reuptake inhibitors (SSRIs) are often prescribed for TN when first-line anticonvulsants such as carbamazepine or oxcarbazepine provide insufficient relief or when comorbid anxiety becomes prominent (46, 47). To determine whether SSRI treatment modulates TN-related behaviors and the activity dynamics of the lPBN-mPF-lPBN positive feedback loop, we systemically administered fluoxetine (10 mg/kg/d, i.p.) for 7 days and evaluated both behavioral and neuronal responses. Fluoxetine treatment substantially alleviated both the sensory and affective dimensions of trigeminal pain in CION mice (Supplemental Figure 10, B–E). Using the same strategy described in Figure 5E, fiber photometry recordings showed that chronic fluoxetine administration markedly reduced calcium signals in lPBN-projecting PF^Glu^ neurons (Supplemental Figure 10, F–I). These findings suggest that serotonergic modulation can influence both TN-related behavioral output and the activity dynamics of the lPBN-mPF-lPBN pathway, highlighting the potential relevance of this network in therapeutic modulation of TN-related symptoms.

### Col25a1 and Syn2 define the PF positive feedback ensemble.

**Figure 6:**
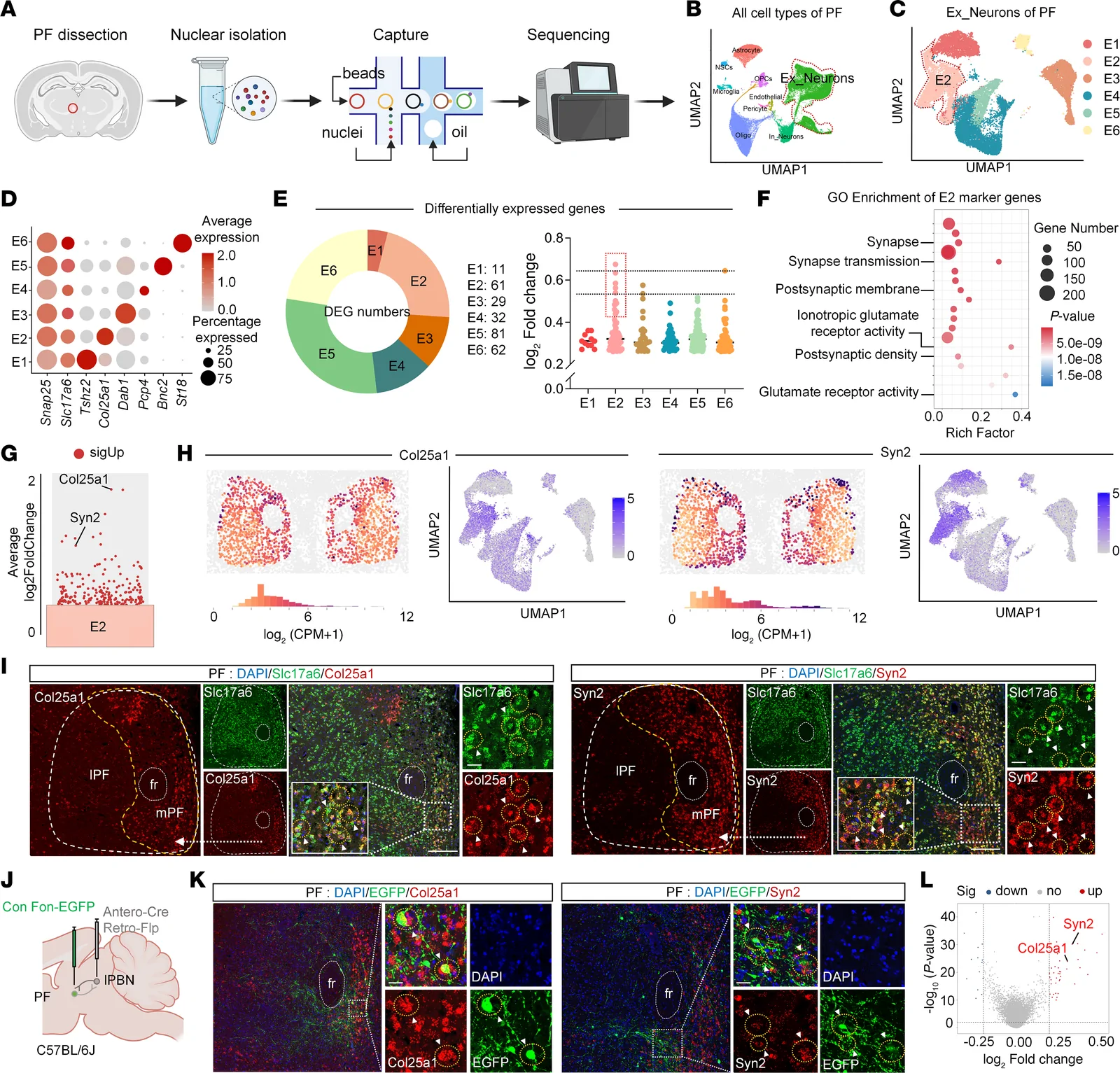
Identification of *Col25a1*/*Syn2* as molecular markers for the PF positive feedback ensemble. (**A**) Schematic outlining of the snRNA-seq experiment. (**B**) Uniform manifold approximation and projection (UMAP) dimensional reduction and visualization of transcriptional profiles of all cell types in the PF, with excitatory neurons (Ex-neurons) encircled by the dashed red line. (**C**) UMAP profiles of Ex-neurons in the PF. (**D**) Dot plot showing expression levels and the proportions of cells expressing specific marker genes in individual PF Ex_neuron clusters. (**E**) Number and log_2_fold-change of DEGs of Ex_neuron clusters. (**F**) GO enrichment scatterplot of E2 marker genes. (**G**) Volcano plot showing expression levels of marker genes in PF E2 cluster, in comparison with all Ex_neuron clusters. (**H**) Feature plots showing expression patterns of *Col25a1* and *Syn2* in the PF. (**I**) RNAscope FISH showing expression of *Col25a1* and *Syn2* in mPF and colocalization with *Slc17a6* (scale bar, 200 μm; zoomed image, 20 μm). (**J** and **K**) Schematic of virus injection (**J**) and representative images of EGFP colocalization with *Col25a1* and *Syn2* in mPF (scale bar, 200 μm; zoomed image, 20 μm) (**K**). (**L**) DEGs of the E2 cluster in sham and CION mice.

To systematically characterize the transcriptional profiles of PF under physiological and TN conditions at the single-cell level, we performed single-nucleus RNA sequencing (snRNA-seq). PF tissues were microdissected from mice 14 days after CION and sham, followed by enzymatic digestion to obtain well-dissociated single nuclei. High-quality nuclei were captured for transcriptomic profiling, and cDNA libraries were subsequently generated for deep sequencing (Figure 6A). Following quality control, we obtained a total of 65,883 single-nucleus transcriptomes, with 34,866 from the sham group and 31,017 from the CION group (Supplemental Figure 11, A, E, and F). Unsupervised clustering revealed 10 major cell clusters for integrated sham and CION groups, each defined by distinct transcriptional signatures (Figure 6B and Supplemental Figure 11, B and D). The clusters were annotated based on the following canonical marker genes: excitatory neurons (*Slc17a6*, *Slc17a7*), inhibitory neurons (*Slc32a1*, *Gad1*, *Gad2*), astrocytes (*Gja1*, *Gfap*), microglia (*Cx3cr1*, *Csf1r*, *Hexb*), oligodendrocytes (*Mog*), committed oligodendrocyte precursor cells (*Fyn*, *Tcf7l2*), oligodendrocyte precursor cells (*Pdgfra*), neural stem cells (*Fam216b*), endothelial cells (*Flt1*), and pericytes (*Rgs5*) (Supplemental Figure 11C).

To further resolve the heterogeneity of excitatory neurons in the PF, we segregated the excitatory neuronal population for in-depth analysis (Figure 6C), which revealed 6 transcriptionally distinct subclusters, each defined by a unique gene expression signature (Figure 6D). The top 3 clusters based on the number of differentially expressed genes (DEGs) induced by CION were E2 (61 genes), E5 (81 genes), and E6 (62 genes) (Figure 6E). Among them, the differences in the expression levels of the DEGs between sham and CION were the most pronounced in the E2 cluster. Besides, E2 cluster had the highest number of DEGs (12 genes) that encode ion channels and receptors (10 genes in E5 cluster and 8 genes in E6 cluster) (Figure 6E and Supplemental Figure 11, G and H). Functional enrichment analysis of the E2 cluster marker genes revealed strong associations with synaptic organization and neurotransmission, alongside pathways known to be linked to neuropsychiatric disorders (Figure 6F and Supplemental Figure 11I). These results suggest that E2 cluster may be the most substantially affected excitatory neuronal subpopulation in the PF on day 14 post-CION.

By integrating the top marker genes (*Col25a1*, *Syn2*, *Thsd7b*, *Kcnt2*, *Tox*, *Necab1*, *Marchf1*, *Kcnt2*, and *Khdrbs2*) of E2 cluster (Figure 6G) with high-resolution spatial transcriptomic data from the Allen Brain Cell Atlas, we found that the DEGs of E2 cluster exhibited location-specific enrichment within the PF. Notably, several key markers, *Col25a1*, *Syn2*, *Kcnt2*, *Khdrbs2*, *Tox*, and *Marchf1*, showed convergent spatial enrichment specifically in the mPF (Figure 6H and Supplemental Figure 11J). These findings suggest that E2 cluster may correspond to the excitatory neuronal population comprising the mPF-localized anxiety-ensemble, with *Col25a1* and *Syn2* serving as potential molecular markers for this functionally defined cell population.

To verify the above prediction, we first validated molecular identity and anatomical localization of the *Col25a1*^+^*Syn2*^+^ subpopulation using RNAscope fluorescence in situ hybridization (FISH) (Figure 6I). Over 90% of *Col25a1*^+^ or *Syn2*^+^ neurons coexpressed *Slc17a6*, confirming their glutamatergic identity. Strikingly, these neurons were predominantly localized within the mPF, consistent with the spatial distribution inferred from the Allen Brain Cell Atlas. To confirm the involvement of this *Col25a1*^+^/*Syn2*^+^ subpopulation in the lPBN-PF-lPBN positive feedback loop, we performed dual in situ hybridization combined with a previously established virus-tracing strategy in Figure 5C to label PF neurons receiving input from and projecting back to the lPBN (Figure 6J). RNAscope FISH for *Col25a1* or *Syn2* was performed after EGFP was fully expressed, enabling precise molecular identification of functionally defined reciprocally connected PF neurons. Most EGFP-labeled neurons expressed *Col25a1* and *Syn2*, confirming that E2 cluster corresponds to the PF anxiety-ensemble (Figure 6K). Differential gene analysis showed that following CION, multiple E2 marker genes, including *Col25a1* and *Syn2*, were significantly upregulated in E2 cluster (Figure 6L), while *Syn2* emerged as a differential gene in all excitatory neuron populations (Supplemental Figure 11L). Functional enrichment analysis revealed that DEGs in E2 cluster were associated with inhibitory synapses, cell adhesion, and neurodegenerative disease pathways (Supplemental Figure 11, M and N, and Supplemental Figure 12A). Together, these findings identify a molecularly distinct PF ensemble localized in the mPF, marked by *Col25a1* and *Syn2*, that constitutes a structural and functional hub of the lPBN-PF-lPBN loop and contributes to pain chronicity of TN.

## Discussion

**Figure 7:**
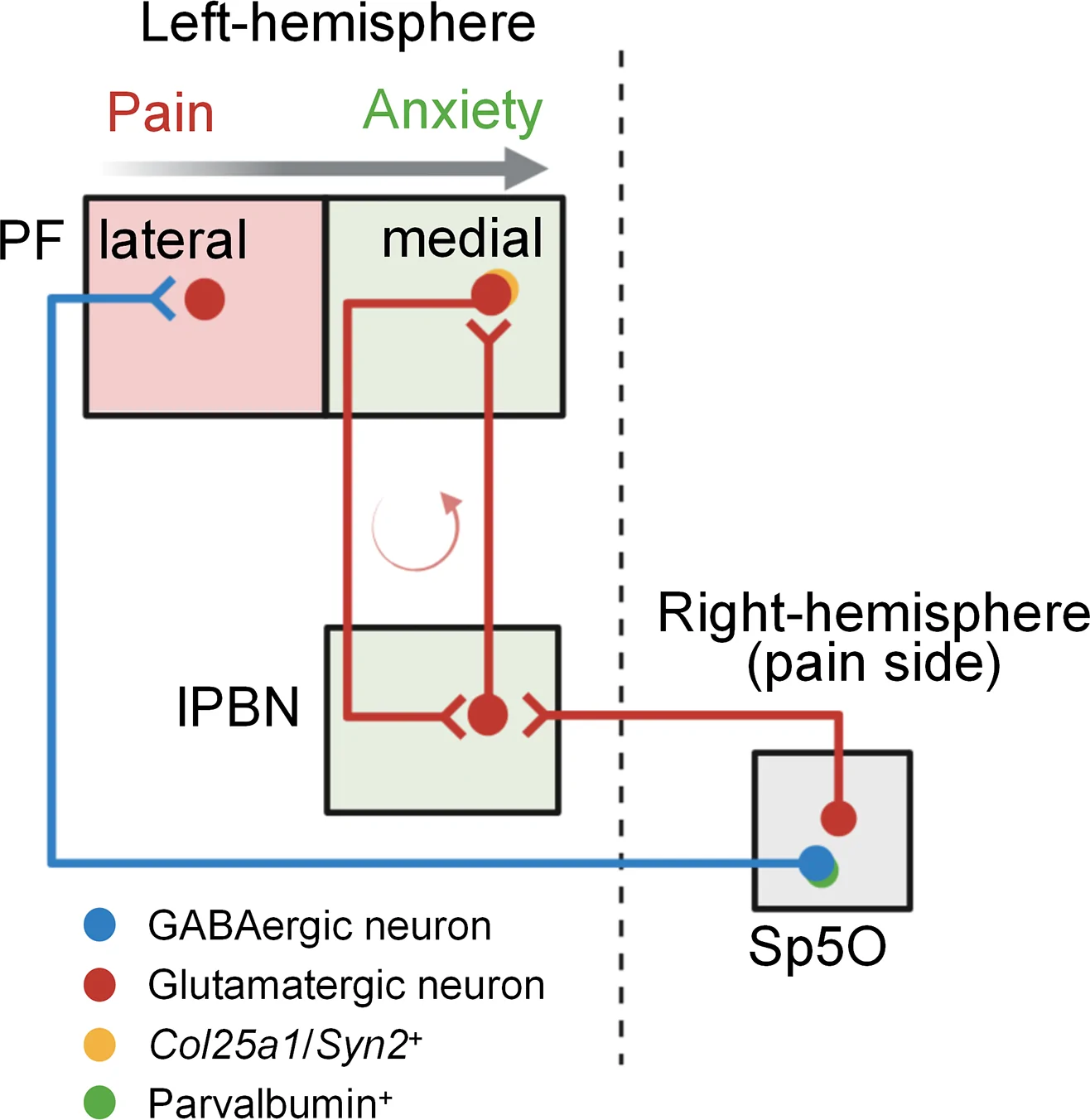
Spatiotemporal role of the PF in TN. PF hyperactivity plays a central role in driving orofacial pain and comorbid anxiety in TN. From upstream, a direct Sp5O^GABA^-PF projection mediates orofacial pain, while enhanced Sp5O^Glu^/lPBN^Glu^/PF transmission encodes pain-related anxiety. These 2 distinct PF neuronal ensembles, defined by their segregated inputs and localization in the lPF and mPF, respectively, contribute to the sensory and affective dimensions of TN. Specifically, the PF anxiety-ensemble engages in a positive feedback loop with the lPBN, and activation of the lPBN/PF/lPBN circuit drives the chronicity of orofacial pain. Moreover, the PF positive-feedback ensemble is characterized by the expression of *Col25a1* and *Syn*2.

TN is among the most excruciating chronic pain disorders, marked by sudden, stabbing, electric shock–like episodes that can be triggered by routine activities. Importantly, TN’s impact extends beyond the sensory manifestations: affective disturbances, such as anxiety, are highly prevalent and substantially worsen patients’ quality of life (1, 48). These challenges underscore the urgent need to elucidate the central mechanisms underlying both the sensory and affective components of TN, which remain poorly understood despite marked clinical relevance. Here, we demonstrate that PF ensembles play a critical role in orchestrating orofacial pain sensation and pain-related anxiety through circuit-level segregation and integration. Sensory and affective dimensions of TN are encoded by separate PF ensembles that receive inputs from Sp5O and PBNsl, respectively. Notably, the mPF anxiety-ensemble innervated by PBNsl also projects back to the PBNsl, forming a reciprocal positive feedback loop, which is recruited later than the PF pain-ensemble and plays a pivotal role in pain chronicity (Figure 7). The identification of *Col25a1* and *Syn2* as molecular markers of this ensemble not only pinpoints a key cellular component of the lPBN-mPF-lPBN loop but also offers potential cell type–specific targets for developing precise and effective therapeutic strategies to manage both orofacial pain and its emotional comorbidities.

As a key component of the intralaminar nuclei, the PF has remained relatively understudied, particularly regarding the topographic organization and functional roles of its subpopulations in nociceptive processing (49). Our findings establish PF as a central node that integrates both the sensory and affective dimensions of orofacial pain, revealing circuitry specificity in orofacial pain and broadening the function of this traditionally “nonspecific” thalamic system (50). In TN, innocuous stimuli can evoke severe pain, reflecting ephaptic cross-excitation between non-nociceptive Aβ and nociceptive Aδ fibers. Projections from Sp5O to the PF provide an ascending pathway that integrates Aβ, Aδ, and C fiber inputs, shaping both the sensory and affective components of orofacial pain (3, 19, 40). Crucially, as the principal upstream subregion directly targeting the PF, the Sp5O also receives direct excitatory inputs from the Sp5C. PF^Glu^ neurons are also known to send collateral projections to several pain- and affect-related regions, such as the central amygdala, nucleus accumbens, and prefrontal cortex, as reported in previous tracing studies (51). These collateral outputs may allow PF to broadcast distinct components of orofacial pain across multiple affective and motivational networks, providing a broader framework for understanding its integrative role in TN circuitry. Together, these findings suggest that PF is uniquely positioned to process trigeminal nociceptive input and affective information, highlighting a system-level specialization in thalamic orofacial pain processing.

The CION model has been extensively used because of its robust and sustained behavioral phenotypes, which enable the investigation of long-term central circuit adaptations and the temporal emergence of TN-related affective comorbidities during chronic pain progression (41, 52). We also note that neurovascular compression–based models, such as the foramen lacerum impingement of trigeminal nerve root (FLIT) model, capture important aspects of classical TN pathophysiology by more closely mimicking vascular impingement of the trigeminal nerve root entry zone. Importantly, recent studies using the FLIT model have demonstrated that decompression and recompression of the trigeminal nerve can bidirectionally and reversibly modulate both pain behaviors and cortical network dynamics, suggesting that central circuit activity in this model remains tightly coupled to ongoing peripheral compression status. Future studies incorporating multiple complementary craniofacial pain models will be important to further capture the heterogeneity of clinical TN and its multidimensional symptomatology.

Although diverse maladaptive plastic changes in both peripheral and central nervous systems have been implicated in the pathogenesis of chronic pain (52–56), the precise definition of pain chronicity and identification of critical windows for therapeutic intervention remain fundamental, yet unresolved, challenges. Accumulating evidence from both clinical and preclinical studies suggests that anxiety often emerges subsequent to pain (1, 41, 43, 45), and the mechanisms underlying nociception and affective disturbances may be at least partially dissociable (57–59). The unique organization of the trigeminal system, which possesses a direct craniofacial-to-lPBN monosynaptic pathway, might preferentially drive affective pain processing (60). Notably, our results showed that female mice develop TN-related sensory and affective phenotypes comparable to males, with a temporally earlier emergence of anxiety-like behaviors and earlier functional recruitment of the PF anxiety-ensemble following CION. These results reveal a sex-dependent temporal pattern, highlighting the need for future studies to dissect the underlying molecular mechanisms of this sex-specific modulation. Taken together, the spatial segregation and sequential engagement of distinct PF neuronal ensembles define a hallmark of pain chronicity, offering a potential framework for understanding the transition from acute to chronic pain.

The architecture of neuronal circuits governs how information is processed and propagated in the brain. A recent study revealed a positive feedback loop involving ACC^Glu^-VTA^GABA^-VTA^DA^-ACC that mediates the persistence of pain and comorbid affective states (61). Using virus tracing, electrophysiology, and optical manipulation, we identified the lPBN-mPF-lPBN circuit as a feedback excitation loop that drives the affective component of pain under the pathological condition of TN. A potential caveat of using mWGA-based viral tracers in reciprocal circuits is their bidirectional trans-synaptic transport property, as mWGA labeling could reflect contributions from both anterograde trans-synaptic transfer and retrograde uptake (38), thereby partially complicating the interpretation of PF-lPBN reciprocal connectivity. Considering this technical limitation, we employed multiple complementary approaches to independently validate the existence of the lPBN-mPF-lPBN positive feedback loop. Structurally, (a) TVA-based retrograde monosynaptic tracing demonstrated that PF-projecting lPBN neurons receive direct feedback input from the mPF (Figure 5A); (b) anterograde tracing independently confirmed the projection from the PF to the lPBN (Figure 5B); and (c) dual-recombinase labeling precisely targeted reciprocally connected mPF neurons, supporting the existence of a closed-loop circuit architecture within the mPF (Figure 5C). Functionally, ex vivo chemogenetic-assisted electrophysiological recording further demonstrated that activation of lPBN-derived axon terminals within the PF region increased the firing frequency of PF neurons that project back to the lPBN (Figure 5G). These findings provide strong convergent evidence supporting the existence of the lPBN-mPF-lPBN positive feedback loop.

Notably, although the Sp5O^Glu^-lPBN^Glu^-PF^Glu^ pathway specifically regulates the pain-related anxiety, manipulation of the positive feedback loop bidirectionally modulates both the orofacial pain and anxiety. Given that mPF neurons not only reciprocally project to PBNsl but also send collateral outputs to PBNdl and PBNel, the reduced mechanical threshold following lPBN-mPF-lPBN loop activation might arise from indirect recruitment of the pain-ensemble via the collateral PBNel-lPF pathway, representing an affective amplification of nociceptive processing. Together, these findings suggest that feedback excitation loops may represent a generalizable principle of circuit organization underlying the pathological status of pain chronicity.

As a key relay for nociceptive signals, the lPBN receives and sends divergent projections to multiple emotion- and instinct-related centers (57, 62–64), enabling it to encode both the sensory and affective dimensions of pain through subregion- and target-specific pathways (58, 65). Continuous chemogenetic activation of either the entire glutamatergic neuronal population (66) or the Calca-expressing glutamatergic subpopulation (67) in the lPBN induces persistent allodynia that remains evident after CNO clearance, resembling the long-lasting pain sensitization produced by chronic activation of the lPBN-mPF-lPBN circuit in our study. Within the lPBN, PBNel plays a key role in mediating mechanical nociceptive thresholds through brainstem reflex arcs that generate rapid exteroceptive defensive responses (68). Although the PF itself is unlikely to participate directly in these reflex circuits, our findings indicate that it exerts descending modulatory control. Specifically, mPF neurons send feedback projections to both the PBNsl and PBNel, regions essential for coordinating orofacial reflex and autonomic responses. Through this descending influence, PF activity can dynamically tune lPBN excitability and thereby regulate the gain of reflexive nocifensive responses.

Through systematic snRNA-seq profiling of the PF under TN and control conditions, we identified marker genes of the E2 cluster that also overlapped with DEGs between the 2 conditions. Although *Syn2* is broadly expressed throughout the central nervous system, both *Syn2* and *Col25a1* exhibited distinct subregional expression patterns within the PF. Importantly, by combining viral tracing strategies with RNAscope FISH, we further demonstrated that *Col25a1^+^* and *Syn2^+^* neurons are highly colabeled with the mPF anxiety-ensemble, suggesting their association with pathological recruitment of the mPF circuit. While *Col25a1* and *Syn2* primarily serve as molecular markers of the mPF anxiety-ensemble in the present study, these findings provide a transcriptional framework for future studies aimed at developing targeted molecular interventions to modulate this cell population and PF-related circuit activity in TN.

In conclusion, we have identified PF as a critical node for orofacial pain regulation, characterized by the spatial segregation and sequential activation of neuronal ensembles in a circuitry- and cell type–specific manner. We further delineate a functional positive feedback loop between the mPF and lPBN that serves as a circuit-level substrate for pain chronicity of TN. Disruption of this loop effectively alleviates both nociceptive and affective symptoms, highlighting it as a promising target for pharmacological or neuromodulatory interventions in the clinical management of orofacial pain.

## Methods

Detailed information on materials and methods is provided in Supplemental Methods.

### Sex as a biological variable.

Our study examined both male and female mice. Age-matched animals of both sexes were studied in parallel across behavioral and electrophysiological experiments. Female mice were included without prescreening for estrous cycle stages to incorporate natural biological variability. Core circuit mechanisms were broadly conserved across sexes, although sex-dimorphic effects in the temporal progression of pain-related affective phenotypes were identified and are reported.

### Animals.

Vglut2-IRES-Cre, Vgat-IRES-Cre, and Pvalb-IRES-Cre transgenic mice were purchased from The Jackson Laboratory. C57BL/6J (20–25 g) mice were supplied by the Animal Center of Peking University, Health Science Center. Bo Peng (Institute for Translational Brain Research, Fudan University, Shanghai, China) provided ROSA26-LSL-tdTomato mice. The animals were housed in a constant temperature of 23 ± 1°C under a 12-hour light/dark cycle. Food and water were available ad libitum. The animals were acclimated for at least 3 days before the beginning of the experimental procedures.

### Statistics.

All experiments were conducted with side-by-side controls and in randomized order. Behavioral testing and subsequent data analysis were performed under double-blind conditions. All data are presented as mean ± SEM unless otherwise indicated. Data shown in box plots (Figures 1, B and C; Figure 3A; and Supplemental Figure 1, A–C) are presented as median with individual data points, and whiskers indicate the minimum and maximum values. Statistical analyses were performed using MATLAB (MathWorks) and Prism 9 (GraphPad Software). For comparisons between 2 groups, either 2-tailed paired or unpaired Student’s *t* tests were used as appropriate. One-way ANOVA followed by the least significant difference test was used for multiple-group comparisons or 2-way ANOVA followed by appropriate post hoc tests when 2 factors were involved. Data normality was assessed using the Shapiro-Wilk test, and homogeneity of variance was evaluated using Levene’s test. Statistical significance was defined as *P* < 0.05. The exact numbers of animals, slices, or cells used for each analysis are provided in the corresponding figures and figure legends.

### Study approval.

All animal care and experimental procedures were approved by the Animal Care and Use Committee of Peking University.

### Data availability.

All data needed to evaluate the conclusions of this study are present in the main paper and/or the supplemental material. Source data for this study are also available in the supplemental Supporting Data Values file. The raw sequence data reported in this paper have been deposited in the Genome Sequence Archive (69) in National Genomics Data Center (70), China National Center for Bioinformation / Beijing Institute of Genomics, Chinese Academy of Sciences (GSA: CRA042101) that are publicly accessible at https://ngdc.cncb.ac.cn/gsa Any additional information required to reanalyze the data reported in this article is available from the lead author upon request.

## Author contributions

CC conceived the study and designed experiments; YL designed studies and performed most experiments and data analysis; YL and YC conducted some of the behavioral experiments and data analysis; YT, YY, and YL performed the analysis of human fMRI; and HLT and YL analyzed the mouse snRNA-seq data. YL performed the electrophysiological recording. YL, HZ, ZH, and QC performed some virus injections and stereotaxic surgery. YL, HZ, and ZH performed some of the optogenetic behavior experiments; JL performed some of the immunochemistry experiments; and CC, HLT, YL, YT, and YW wrote the manuscript. All authors reviewed the manuscript and approved its submission.

## Conflict of interest

The authors have declared that no conflict of interest exists.

## Funding support

- National Key Technology Support Program of the Ministry of Science and Technology of China (2021ZD0203204 to YW).
- National Natural Science Foundation of China (32571184 to CC).
- National Natural Science Foundation of China Young Student Basic Research Program (for PhD students) (324B2030 to YL).
- National Key Technology Support Program of the Ministry of Science and Technology of China (2022ZD0206400 to YT).
- Beijing Natural Science Foundation (5242008 to CC).
- Clinical Medicine Plus X-Young Scholars Project of Peking University (PKU2022LCXQ032 to CC).

## Supplementary Material

Supplemental data

Supporting data values

## Version 1 07/14/2026

In-Press Preview

## Version 2 09/01/2026

Electronic publication

## Version 3 09/03/2026

Corrected color shading in figures 2J-Q and 4N-Y
