# Supplementary material for: Distinct parafascicular neuronal ensembles mediate the sensory and affective dimensions of trigeminal neuralgia
Source: J Clin Invest. 2026 Jul 14;136(17):e206862. doi: 10.1172/JCI206862 (PMC13528938; doi:10.1172/JCI206862)
Supplement: Supplementary file 1 [file jci-136-206862-s008.pdf]

Supplemental Methods

Human Neuroimaging Data Acquisition and Processing

Neuroimaging data were obtained from the open-access dataset ds005713 (version 1.0.1) from the OpenNeuro repository (https://open-neuro.org/datasets/ds005713/versions/1.0.1). MRI data were acquired at two time points: pre-operatively and 6 months post-operatively. For each T1-weighted image, fully automated surface-based morphometric analysis was conducted using FreeSurfer v7.1.1 software (https://surfer.nmr.mgh.harvard.edu). The standard "recon-all" pipeline was employed, which included motion correction, intensity normalization, skull stripping, linear and nonlinear volumetric registrations, spherical surface registration, subcortical segmentation, and cortical parcellation based on the Desikan-Killiany atlas. Intracranial volume (ICV) was extracted from the FreeSurfer outputs for use as a covariate in subsequent statistical analyses. Following this, thalamic nuclei segmentation was performed using a previously validated probabilistic algorithm (1). Representative thalamic nuclei segmentation and the PF segmentation from one subject are illustrated in Figure 1A.

CION surgery

Chronic constriction injury of the infraorbital nerve (CION) was performed via an intraoral approach to establish the TN model, as previously described (2). Briefly, mice were anesthetized with 1.25% Avertin (tribromoethanol, 0.2 ml/10 g, i.p.) and placed in the supine position. A small incision was made along the left gingivobuccal margin as

viewed from the surgeon's position, approximately 1 mm posterior to the first molar. The infraorbital nerve was exposed and loosely ligated with a single 4-0 polyglycolic-co-lactic acid suture. Sham controls underwent nerve exposure without ligation. All procedures were conducted under sterile conditions.

SNI surgery

The spared nerve injury (SNI) model was established following a previously reported protocol (3). In brief, mice were anesthetized with 1.25% Avertin (tribromoethanol, 0.2 ml/10 g, i.p.), and the sciatic nerve was exposed near the thigh. The sural, common peroneal, and tibial branches were identified and separated. The common peroneal and tibial nerves were tightly ligated at the trifurcation using 4-0 silk sutures, then transected distal to the ligature, with 3-5 mm of the distal segments removed. The sural nerve remained intact throughout the procedure. In the sham group, the sciatic nerve was similarly exposed but without ligation or transection of its branches.

Stereotaxic surgery

During stereotactic surgery, mice were anesthetized with isoflurane (4% for induction and 1.5% for maintenance) and securely positioned in a stereotactic apparatus (RWD Life Science, Shenzhen, China). A volume of 0.06-0.08 μL of virus was unilaterally injected at the target site at a flow rate of 0.05 μL/min. To ensure proper diffusion and minimize the risk of backflow, a 5-minute waiting period was observed following each injection. The stereotaxic coordinates for injections were as follows: PF: AP −2.25 mm,

ML −0.68 mm, DV −3.65 mm; lPBN: AP −5.20 mm, ML +1.25 mm, DV −3.30 mm; and Sp5O: AP −6.0 mm, ML +1.7 mm, DV −5.2 mm. The pipette was allowed to remain in place for another 10 min after injection, leaving the virus well absorbed before retraction. Following surgery, mice were placed on a heating pad until fully awake and then returned to their home cages. Three weeks later, when required, guide cannulas (RWD Instruments) or optical fibers (Inper) were implanted into the indicated site (PF). Cannulas and optical fibers were secured to the skull with skull screws and dental cement.

Virus construction and injection

For anterograde and retrograde tracing of the PF, rAAV-hSyn-EGFP-WPRE-hGH (AAV2/Retro, 5.0×10^{12} vg/ml) was injected into the PF of C57BL/6J mice, rAAV-hSyn-SV40 NLS-Cre (AAV2/1, 1.2×10^{13} vg ml^{-1}) was injected into the PF of Ai14 mice. For retrograde tracing of the PF-projecting $Sp5O^{PV}$ neurons, rAAV-hSyn-SV40 NLS-Flp-P2A-mCherry (AAV2/Retro, 5.0×10^{12} vg ml^{-1}) was injected into the PF and rAAV-hSyn-Con Fon-EYFP (AAV2/9, 5.0×10^{12} vg ml^{-1}) was injected into the Sp5O. For retrograde tracing of Sp5O neurons that project to the PF via lPBN, rAAV-EF1α-DIO-Flp (AAV2/Retro, 5.0×10^{12} vg ml^{-1}) and CTB488 were injected into the PF, rAAV-EF1α-fDIO-EGFP-T2A-TVA (AAV2/9, 5.0×10^{12} vg ml^{-1}) and rAAV-EF1α-fDIO-N2cG (AAV2/9, 5.0×10^{12} vg ml^{-1}) were injected into the lPBN. Then, CVS-EnvA-ΔG-tdTomato (2.0×10^{8} infectious units per ml) was injected into the lPBN three weeks later. For anterograde tracing of Sp5O and PBNsl projection subregions within

the PF, rAAV-hSyn-SV40 NLS-Cre (AAV2/1, 1.2 × 10^{13} vg ml^{-1}) was injected into the Sp5O, rAAV-CaMKIIα-mWGA-EGFP (AAV2/9, 5.0 × 10^{12} vg ml^{-1}) was injected into the PBNsl and a Cre-dependent reporter virus rAAV-hSyn-DIO-mCherry (AAV2/9, 5.0 × 10^{12} vg ml^{-1}) was delivered into the PF. For anterograde and retrograde tracing of the PF anxiety-ensemble, rAAV-hSyn-SV40 NLS-Cre (AAV2/1, 1.2 × 10^{13} vg ml^{-1}) and rAAV-hSyn-SV40 NLS-Flp-P2A (AAV2/Retro, 5.0 × 10^{12} vg ml^{-1}) were injected into the lPBN, and rAAV-hSyn-Con Fon-EYFP (AAV2/9, 5.0 × 10^{12} vg ml^{-1}) was injected into the PF.

For optical fiber photometry of PF^{Glu} neurons, rAAV-EF1α-DIO-GCaMP6s-WPRE-hGH (AAV2/9, 5.0 × 10^{12} vg ml^{-1}) was injected into the PF of Vglut2-Cre mice. For optical fiber photometry of different PF ensembles that receive the Sp5O or the lPBN inputs, rAAV-CAG-DIO-mWGA-FLP-WPRE-hGH poIyA (AAV2/9, 5.0 × 10^{12} vg ml^{-1}) was injected into the Sp5O or the lPBN, and rAAV-nEF1a-fDIO-GCaMP6s-mCherry-WPRE-hGH (AAV2/9, 5.0 × 10^{12} vg ml^{-1}) or rAAV-nEF1a-fDIO-GCaMP6s-WPRE-hGH (AAV2/9, 5.0 × 10^{12} vg ml^{-1}) was injected into the PF. Three weeks later, an optical fiber (200 μm, NA = 0.37; Inper, Hangzhou, China) was implanted into the PF for photometry recordings. For functional connections of the $lPBN^{Glu}$-PF^{Glu}-$lPBN^{Glu}$ feedback loop, rAAV-CAG-DIO-mWGA-FLP-WPRE-hGH poIyA (AAV2/9, 5.0 × 10^{12} vg ml^{-1}) was injected into the PF, rAAV-nEF1a-fDIO-ChrimsonR-WPRE-hGH polyA (AAV2/9, 5.0 × 10^{12} vg ml^{-1}) and rAAV-hSyn-GCaMP6s-WPRE-hGH polyA (AAV2/Retro, 5.0 × 10^{12} vg ml^{-1}) into the lPBN, Three weeks later, an optical fiber (200

μm, NA = 0.37; Inper, Hangzhou, China) was implanted into the PF for photometry recordings.

For optogenetic manipulation of PF^{Glu} neurons, rAAV-EF1a-DIO-eNpHR3.0-EGFP-WPRE-hGH polyA (AAV2/9, 5.0 × 10^{12} vg ml^{-1}) or rAAV-EF1a-DIO-hChR2(H134R)-EGFP-WPRE-hGH polyA (AAV2/9, 5.0 × 10^{12} vg ml^{-1}) was injected into the PF of Vglut2-Cre mice. Three weeks later, an optical fiber (Inper) was implanted into the PF. rAAV-EF1a-DIO-eNpHR3.0-EGFP-WPRE-hGH polyA (AAV2/9, 5.0 × 10^{12} vg ml^{-1}) was injected into the Sp5O for optogenetic inhibition of $Sp5O^{Glu}$-PF circuit, while rAAV-EF1a-DIO-hChR2(H134R)-EGFP-WPRE-hGH polyA (AAV2/9, 5.0 × 10^{12} vg ml^{-1}) was injected into the Sp5O for optogenetic activation of $Sp5O^{PV}$-PF circuit. For inhibition of the $Sp5O^{Glu}$-$lPBN^{Glu}$-PF circuit, rAAV-CAG-DIO-mWGA-FLP-WPRE-hGH poIyA (AAV2/9, 5.0 × 10^{12} vg ml^{-1}) and CTB488 were injected into the Sp5O, rAAV-nEF1a-fDIO-eNpHR3.0-mCherry-WPRE-hGH polyA (AAV2/9, 5.0 × 10^{12} vg ml^{-1}) or rAAV-nEF1a-fDIO-mCherry-WPRE-hGH polyA (AAV2/9, 5.0 × 10^{12} vg ml^{-1}) was injected into the lPBN of Vglut2-Cre mice. Three weeks later, an optical fiber (Inper) was implanted into the PF. For inhibition of the PF terminals in lPBN that receive inputs from $lPBN^{Glu}$ neurons, rAAV-CAG-DIO-mWGA-FLP-WPRE-hGH poIyA (AAV2/9, 5.0 × 10^{12} vg ml^{-1}) was injected into the lPBN and rAAV-nEF1a-fDIO-eNpHR3.0-mCherry-WPRE-hGH polyA (AAV2/9, 5.0 × 10^{12} vg ml^{-1}) into the PF. Three weeks later, an optical fiber (Inper) was implanted into the lPBN.

For chemogenetic manipulation of PF^{Glu} neurons, rAAV-EF1a-DIO- hM3D(Gq)-EGFP-WPRE-hGH polyA (AAV2/9, 5.0 × 10^{12} vg ml^{-1}) or rAAV-EF1a-DIO-hM4D(Gi)-EGFP-WPRE-hGH polyA (AAV2/9, 5.0 × 10^{12} vg ml^{-1}) was injected into the PF of Vglut2-Cre mice to facilitate neuronal activation or inhibition, respectively. Control groups received injections of rAAV-DIO-EGFP-WPRE-hGH (AAV2/9, 5.0 × 10^{12} vg ml^{-1}) into the PF.

For electrophysiological recording of the Sp5O^{GABA}-PF pathway, rAAV-EF1a-DIO-hChR2(H134R)-EGFP-WPRE-hGH polyA (AAV2/9, 5.0 × 10^{12} vg ml^{-1}) was injected into the Sp5O of Vgat-Cre mice. For electrophysiological recording of the Sp5^{Glu}-lPBN^{Glu}-PF pathway, rAAV-CAG-DIO-mWGA-FLP-WPRE-hGH poIyA (AAV2/9, 5.0 × 10^{12} vg ml^{-1}) was injected into the Sp5O, and rAAV-nEF1a-fDIO-hChR2(H134R)-mCherry-WPRE-hGH polyA (AAV2/9, 5.0 × 10^{12} vg ml^{-1}) was injected into the lPBN of Vglut2-Cre mice. For electrophysiological recording of lPBN-projecting PF neurons in response to chemogenetic activation of the lPBN^{Glu}-PF pathway, rAAV-EF1α-mCherry-WPRE-hGH polyA (AAV2/Retro, 5.0 × 10^{12} vg/ml) and rAAV-EF1α-DIO-hM3D(Gq)-EGFP-WPRE (AAV2/9, 5.0 × 10^{12} vg/ml) were injected into the lPBN of Vglut2-Cre mice. Four weeks later, the mice were sacrificed for patch-clamp recording. All viruses were from Brain Case or BrainVTA.

Slice preparation and electrophysiological recording

Acute coronal brain slices (300 μm) containing the PF were prepared from male and

female C57BL/6J or Vglut2-Cre mice. Animals were anesthetized with 1.25% Avertin (tribromoethanol, 0.2 ml/10 g, i.p.), decapitated, and the brains were rapidly removed. Slicing was performed in ice-cold, oxygenated cutting solution containing (in mM): 213 sucrose, 26 $NaHCO_3$, 1 NaH_2PO_4, 3 KCl, 0.5 $CaCl_2$, 5 $MgCl_2$, and 10 glucose (pH 7.4, 290-320 mOsm), which was continuously bubbled with 95% O_2 / 5% CO_2. Slices were then transferred to artificial cerebrospinal fluid (ACSF) containing (in mM): 125 NaCl, 3 KCl, 1.2 NaH_2PO_4, 26 $NaHCO_3$, 0.5 $CaCl_2$, 5 $MgCl_2$, and 10 glucose (pH 7.4, 290-320 mOsm), bubbled with 95% O_2 / 5% CO_2 and incubated at 37°C for at least 1 h prior to recording. Recording pipettes were pulled using a horizontal puller (P97, Sutter Instruments, Novato, CA, USA) and had a resistance of 4-8 MΩ when filled with the pipette solution. For current-clamp recordings and excitatory synaptic events, the pipette solution contained (in mM): 140 K-gluconate, 8 NaCl, 2 $MgCl_2$, 2 Na_2ATP, 0.3 NaGTP, 1 EGTA, and 10 HEPES (pH 7.3-7.4, adjusted with KOH; 290-320 mOsm). For inhibitory synaptic current recordings, the pipette solution consisted of (in mM): 110 CsCl, 30 K-gluconate, 5 NaCl, 2 $MgCl_2$, 10 HEPES, 0.5 EGTA, 2 ATP, and 0.4 GTP (pH 7.3-7.4, adjusted with KOH; 290-320 mOsm).

A slice was viewed with an upright microscope (Axioskop Fsmot, Zeiss, Germany) equipped with infrared-differential interference contrast optics. Cells of the PF were easily recognizable via a 40x water-immersion lens. Voltage and current signals were recorded from PF neurons using an EPC10 patch clamp amplifier (HEKA Instruments). The APs were recorded using the current-clamp mode. For sEPSC recordings,

picrotoxin (50 mM) was added to block $GABA_A$ receptor-mediated inhibitory synaptic currents. For sIPSC recordings, CNQX (10 μM) and APV (50 μM) was added to block AMPA and NMDA receptor-mediated excitatory synaptic currents. sEPSCs and sIPSCs were recorded using the voltage-clamp mode, with the neuron voltage-clamped at -70 mV. Series resistance (Rs) was monitored at regular intervals throughout the recording experiment. Cells with Rs larger than 25 MΩ during the recording were discarded. Data were acquired and analyzed with the EPC10 patch clamp amplifier and Clampfit 10.7 (Molecular devices, USA). The analysis of sEPSCs and sIPSCs was performed blindly using MiniAnalysis software (Synaptosoft). The average amplitude and frequency of sEPSCs and sIPSCs were determined by the two-tailed t-tests. 50 random points selected from each cell were concatenated to describe the cumulative distributions of sEPSCs and sIPSCs in each condition and then compared by a Kolmogorov-Smirnov two-sample test.

Optical stimulation (589 nm, continuous; or 473 nm at 5 Hz) was delivered using laser generators (Newdoon, Hangzhou, China) through an optical fiber 200 μm in diameter and positioned ~200 μm from the surface of the slice. oEPSCs or oIPSCs were recorded in voltage-clamp mode (holding potential, −70 mV for oEPSCs and oIPSCs), with the K-gluconate- or CsCl-based pipette solution, respectively, as described above. To record Sp5O-PF synaptic transmission, light was delivered to Sp5O GABA-positive terminals expressing ChR2 in the PF of Vgat-Cre mice. To record the PPR, paired optical stimuli (473 nm, 5 mW, 5 ms, interpulse interval 100 ms) were applied, and the

oEPSC2/oEPSC1 ratio was calculated. To record the glutamatergic synaptic transmission between lPBN^{Glu} neurons and PF^{Glu} neurons, light was delivered to lPBN^{Glu} terminals expressing ChR2 in the PF of Vglut2-Cre mice. To test whether the postsynaptic currents recorded in PF neurons were elicited by direct Sp5O synaptic connections, 1 μM TTX and 100 μM 4-AP were added to ACSF.

Chemogenetic and optogenetic manipulation *in vivo*

For chemogenetic experiments, mice underwent behavioral testing 3-4 weeks after viral injection to allow for sufficient transgene expression. CNO (1 mg/kg, dissolved in saline; Selleck, Shanghai, China) or saline was administered via intraperitoneal injection 30 minutes prior to the onset of behavioral tests.

Two weeks after injection of optogenetic viruses, optical fibers (Ø200-μm core, 0.37 numerical aperture; Inper, Hangzhou, China) were implanted 0.2 mm above the virus injection site under anesthesia. Mice were allowed to recover for 1 week. Before optogenetic stimulation, the laser source was adjusted to the proper intensity and connected to the implanted optical fiber through a fiber cable. von Frey test, EPM test, and OFT were performed with yellow light stimulation (589 nm, 10 mW, continuous).

Chemogenetic Drug Delivery

To locally modulate neural activity, Clozapine N-oxide (CNO; 3 μM, Sigma-Aldrich) dissolved in sterile 0.9% saline containing 0.5% DMSO was microinfused into the PF.

A stainless-steel injector (outer diameter: 0.2 mm; inner diameter: 0.1 mm; RWD Life Science, Shenzhen, China) was connected to a 1 μL microsyringe (Hamilton, Switzerland) via PE tubing filled with mineral oil, creating a sealed system. The injector was carefully inserted into the pre-implanted guide cannula, and a total volume of 0.1 μL CNO solution was infused into the PF at a flow rate of 0.05 μL/min. To ensure proper diffusion and minimize backflow, the injector was left in place for an additional 5 minutes following the injection. The guide cannula was then recapped and secured. Mice were returned to their home cages and allowed to rest for 15 minutes to allow the drug to equilibrate before undergoing behavioral tests. Control mice received an identical microinfusion of the vehicle alone.

Optical fiber photometry

Calcium recordings in behaving animals were conducted using fiber photometry (three colors) with optogenetics (QAXK-FPS-SS-LED-CH, Thinker Tech, Nanjing, China). In brief, rAAV-CAG-DIO-mWGA-FLP-WPRE-hGH polyA was injected into the PF, and rAAV-nEF1a-fDIO-ChrimsonR-WPRE-hGH polyA and rAAV-hSyn-GCaMP6s-WPRE-hGH were injected into the lPBN. The mice were allowed 1 week to recover before Ca^{2+} signal recording. The 470 nm and 580 nm laser beams were reflected off a dichroic mirror and focused with an objective lens to excite GCaMP6s and ChrimsonR channels, respectively. The relative change in fluorescence, ΔF/F, was calculated using the formulas: $\Delta F/F = (F_T - F_0) / (F_0 - F_{offset})$, where F_T is the mean fluorescence during the event-related period, F_0 is the mean fluorescence during the baseline period, and

F_{offset} is the background fluorescence signal. The 405-nm channel was used as a reference for calcium signal correction. Event onset (zero point) was defined as the time of behavior stimulation, and calcium signal data from −2 to +10 seconds relative to the event were analyzed using MATLAB (2023a). Results were presented as heat maps, peri-event plots, and area under the curve (AUC) plots.

Immunohistochemistry

Mice were first anesthetized with 1.25% Avertin (tribromoethanol, 0.2 ml/10 g, i.p.), followed by transcardial perfusion with phosphate-buffered saline (PBS), and subsequently with 4% paraformaldehyde (PFA). After perfusion, brains were carefully extracted and post-fixed overnight in 4% PFA. The brains were then immersed sequentially in 10%, 20%, and 30% sucrose buffers, with each step lasting 24 hours at 4°C, until the tissue sank to the bottom. The tissue was embedded in Tissue-Tek Optimum Cutting Temperature (OCT) compound (Sakura Finetek, USA), and coronal sections of 40 μm thickness were cut using a cryostat microtome (CM1950, Leica, Germany).

For immunohistochemical staining, brain sections were first incubated in a blocking buffer (3% bovine serum albumin, 0.3% Triton X-100 in PBS) for 1 hour at room temperature. The sections were then incubated with primary antibodies overnight at 4°C. After washing with PBS, sections were incubated with secondary antibodies for 2 hours at room temperature. The stained sections were mounted on slides (2–6 slices per slide)

and covered with mounting medium containing DAPI (Solarbio Life Science, China). Confocal images were captured using a confocal microscope (FV3000, Olympus, Japan).

The primary antibodies included: Mouse anti-GAD67 (1:500, MAB5406, Merck, USA) and Rabbit anti-Glutamate (1:500, G6642, Merck, USA). Secondary antibodies included: Goat anti-rabbit Alexa Fluor 488 (1:1000, A-11034, Invitrogen, USA) and Goat anti-rabbit Alexa Fluor 594 (1:1000, A-11012, Invitrogen, USA).

For tissue clearing, post-fixed brain tissue was processed using a tissue clearing kit (Nuohai Life Science, Shanghai, China). The tissue was sequentially immersed in hydrophilic solutions for 3-5 days to achieve delipidation, followed by 2 days of refractive index matching. The procedure was complete when background patterns could be clearly visualized without distortion. The tissue was then embedded in gel and solidified at 4°C for 2 hours before imaging with a light sheet microscope (LS18, Nuohai Life Science, Shanghai, China). Image analysis was performed using AIVIA software.

Fluorescence in situ hybridization

Multiplex fluorescent in situ hybridization was performed using the RNAscope system (Advanced Cell Diagnostics, ACD) following the manufacturer’s protocol. Briefly, brain sections were sequentially pretreated with hydrogen peroxide, target retrieval

solution, and Protease III. Sections were then hybridized for 2 h at 40°C with target probes against vGlut1 (ACDBio, 416631). Signal amplification was achieved through sequential incubation with AMP1, AMP2, and AMP3 (15-30 min each), followed by horseradish peroxidase (HRP)-conjugated detection reagents for channel 1, 2, or 3 (HRP-C1/C2/C3; 15 min). Fluorescent labeling was performed using Opal dyes (Opal 520, 570, or 690; 1:1000; PerkinElmer) for 30 min. After each round of fluorophore development, HRP blocker was applied for 15 min to prevent cross-channel labeling. All hybridization and incubation steps were carried out at 40°C in a humidified hybridization oven. To preserve native EGFP signals, which are easily quenched during the RNAscope procedure, RNAscope was performed on 15-μm brain sections adjacent to those used for direct EGFP imaging.

Behavioral assessment

All behavioral tests were conducted during the light cycle (7:00-19:00). Prior to formal testing, mice were acclimated to the test environment for 30 minutes per day over three consecutive days. Data analysis was performed by researchers blinded to the experimental groups.

Orofacial von Frey test

Mice were handled by the experimenter for 10 min per day for at least 3 consecutive days prior to behavioral testing to minimize handling-induced stress. Mechanical threshold in the infraorbital territory was assessed using von Frey filaments (Danmic,

USA) and the up-down method. Mice were gently restrained by the experimenter wearing a soft leather glove to minimize stress. Filaments were applied perpendicularly to the skin near the center of the vibrissa pad (V2 territory). A brisk head withdrawal or escape reaction was scored as a positive response. Testing began with a mid-range filament (0.4 g for CION mice), and subsequent stimuli were delivered in ascending or descending order based on the response, following up-down paradigm. The 50% withdrawal threshold was calculated as described (4).

Non-evoked nociceptive behavior

CION produces ongoing, non-evoked discomfort in the orofacial region innervated by the injured infraorbital nerve (5, 6). To quantify spontaneous facial grooming, mice were recorded in a standard open-field arena, and video traces were analyzed offline. After a period of acclimation, the cumulative duration of forepaw-to-face contact (including rubbing of the vibrissal and perinasal regions) was measured to assess spontaneous nociceptive behavior.

Conditional place preference

On the pre-conditioning day, mice were placed in a three-chamber apparatus (20 × 50 × 10 cm) for 20 min and allowed to freely explore all compartments to assess baseline preference. Across the following 3 days, animals underwent daily conditioning sessions in which they were confined to one chamber associated with the stimulation condition for 20 min, and to the alternate chamber for an equivalent 20-min session without

stimulation. On the test day, mice were returned to the apparatus and allowed unrestricted access to all chambers, and their chamber occupancy was recorded to assess preference.

Open-field test

Anxiety-like behavior was assessed in an open field apparatus (AniLab, Ningbo, China) consisting of a white plastic chamber (50 × 50 × 50 cm) under ambient lighting (100-200 lux). Mice were placed in the center of the arena and allowed to explore freely for 5 min. Behavior was recorded using an infrared video tracking system, and parameters including total distance traveled and time spent in the center zone were analyzed using SMART software (v2.5.21, Panlab). Reduced center time was interpreted as increased anxiety-like behavior.

Elevated plus maze

The elevated plus maze (EPM) consisted of two open arms (30 × 5 cm) and two closed arms of the same size, enclosed by 14 cm-high side and end walls. The maze was elevated 74 cm above the floor, and a central square (5 × 5 cm) connected all four arms. Mice were placed in the center facing a closed arm and allowed to explore for 5 min. The time spent in open arms and the number of open arm entries were recorded as measures of anxiety-like behavior. Open arm entries and open arm time were analyzed with SMART software (v2.5.21, Panlab). The apparatus was cleaned with 75% ethanol between trials.

Single nuclei isolation and snRNA-seq

SnRNA-seq of the mouse PF was performed by LC-Bio Technologies (Hangzhou, China) using a modified protocol based on previously published methods (7). Briefly, dissected PF tissues were rapidly frozen and stored at –80 °C until processing. For nuclei extraction, frozen PF samples were minced on ice and incubated in 1 ml of lysis buffer (10 mM Tris-HCl, 10 mM NaCl, 3 mM $MgCl_2$, 0.1% NP-40, 0.1 mM $CaCl_2$, and RNase inhibitor) for 7 minutes. The lysis was quenched by adding an equal volume of salt-Tris buffer (250 mM sucrose, 25 mM KCl, 5 mM $MgCl_2$, 20 mM Tris-HCl, pH 7.4), followed by gentle pipetting. The lysate was filtered through a 40-μm cell strainer (BD Falcon) to remove tissue debris, transferred to a 15 ml conical tube, and centrifuged at 500 × g for 5 minutes at 4 °C. The pellet containing nuclei was washed twice in a wash buffer containing 1% bovine serum albumin and RNasin (Promega), and resuspended in 100 μl of the same buffer. Nuclei quality and concentration were assessed by trypan blue staining and counted using a hemocytometer. The suspension was diluted to a final concentration of 700~1200 nuclei/μl.

Approximately 10,000 nuclei were loaded into each channel of the Chromium controller (10x Genomics) for barcoding and droplet generation, using the Chromium Next GEM Single Cell 3' Reagent Kits v3.1 (10x Genomics, 1000268). Libraries were prepared using the Chromium Single Cell 3'/5' Library Construction Kit (10x Genomics, 1000020) according to the manufacturer’s protocol. Final libraries were sequenced on

an Illumina NovaSeq 6000 platform to achieve high coverage. Raw sequencing data were processed using Cell Ranger software (v3.1.0, 10x Genomics), including demultiplexing, alignment to the mm10 mouse reference genome using STAR, and generation of the gene-barcode matrix for downstream analyses.

snRNA-seq data analysis

Downstream analyses were carried out using the Seurat v4 package implemented in R (8). A common concern in droplet-based snRNA-seq is the inclusion of low-quality nuclei and multiplets. To mitigate this, we first filtered out nuclei whose UMI or gene counts deviated more than two standard deviations from the mean, assuming a Gaussian distribution for these features. Additionally, nuclei with high mitochondrial transcript content were excluded based on a predefined threshold, following visual inspection of the distribution of mitochondrial gene percentages. Doublets were identified and removed using the DoubletFinder package (9). Library size normalization was conducted in Seurat to acquire the normalized count after filtering. Sample integration was accomplished through canonical correlation analysis reduction (10), with the 2000 most variably expressed genes in each sample, identified via a variance-stabilizing transformation, serving as anchor features.

For clustering, the integrated dataset was scaled and centered prior to dimensionality reduction via principal component analysis (PCA). The top 30 principal components (PC1-PC30) were used to construct a shared nearest-neighbor graph, followed by

Louvain clustering (resolution set at 0.8) to identify distinct cellular populations. UMAP visualization was performed using the same set of principal components. Cluster-specific marker genes were identified using the FindAllMarkers function in Seurat, comparing each cluster to all others. Known canonical markers were then used to classify major cell types. To further resolve neuronal subpopulations, clusters identified as neurons were segregated and reprocessed for independent dimensionality reduction, reclustering, and differential expression analysis.

Supplemental Figures and Legends

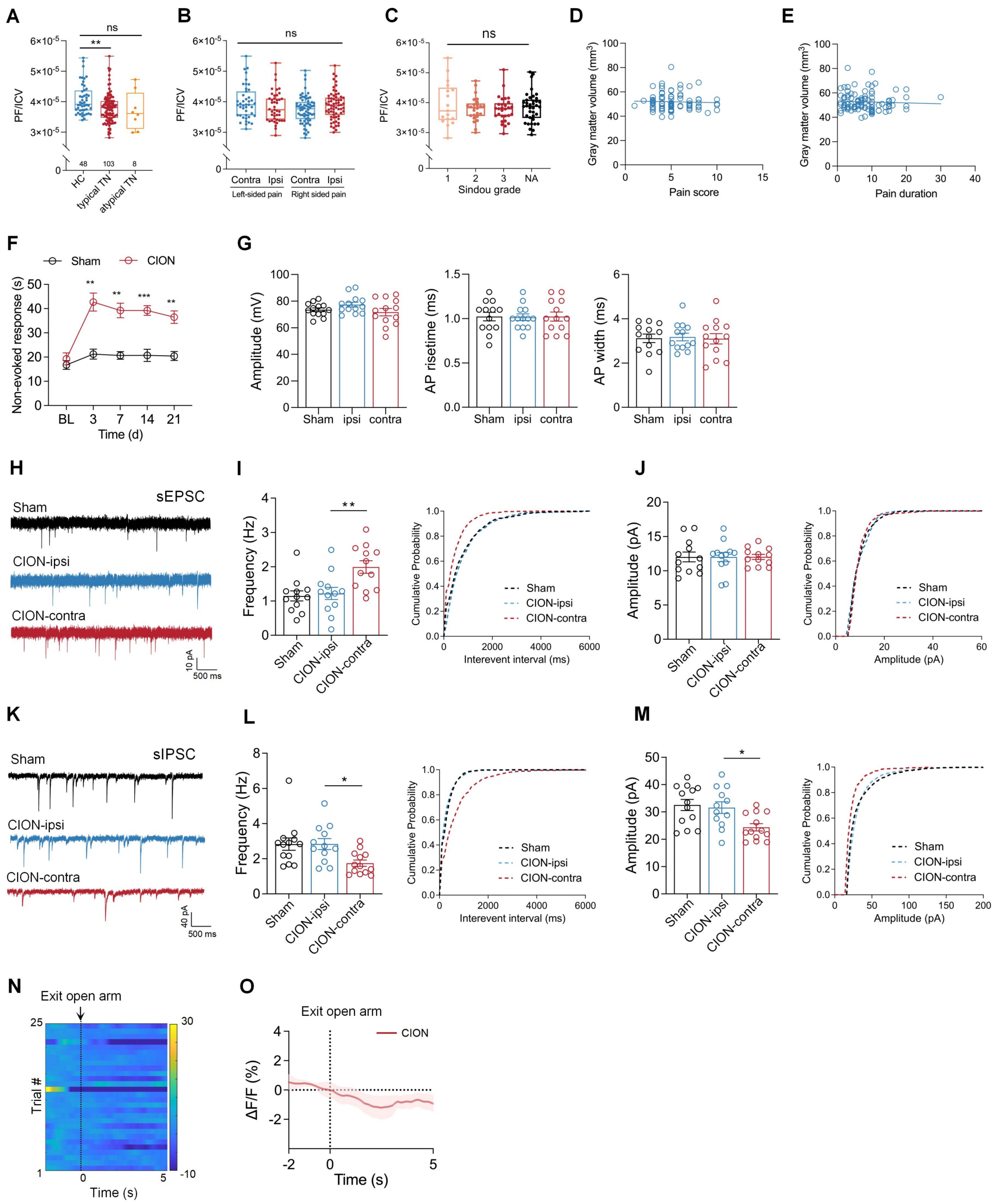

Supplemental Figure 1. CION induces excitation-inhibition imbalance in the PF from upstream synaptic transmission.

A, Ratio of PF gray matter volume to intracranial volume in HC, typical TN and atypical TN patients. **B**, Comparison of the ratio of PF gray matter volume in TN patients of different pain sides. **C**, Comparison of the ratio of PF gray matter volume to intracranial volume based on Sindou grade. **D-E**, Correlation plots displaying no significant relationship between pain score (**D**)/duration (**E**) and the PF gray matter volume. **F**, Non-evoked responses of Sham and CION mice on different days (n = 8 mice for each group). **G**, AP firing amplitude, risetime, and width in Sham, CION-ipsi, and CION-contra PF^{Glu} neurons. **H-J**, Patch clamp recording of sEPSCs showing an increased

frequency of sEPSCs, characterized by a left-shifted cumulative fraction, in CION-contra PF^{Glu} neurons, with no changes in sEPSC amplitude. **K-M**, Patch clamp recording of sIPSCs showing decreases in both frequency and amplitude of sIPSCs in CION-contra PF^{Glu} neurons. **N-O**, Heat maps (**N**), averaged normalized traces of changes (**O**) in GCaMP6s signals when CION mice moved out of open arm in EPM. Data are presented as means ± s.e.m. One-way ANOVA with Tukey's post-hoc test for **A**, **B**, **C**, **G**, **I**, **J**, **L**, **M**, and two-way ANOVA with Tukey's post hoc test for **F**. **P* < 0.05, ***P* < 0.01, ****P* < 0.001.

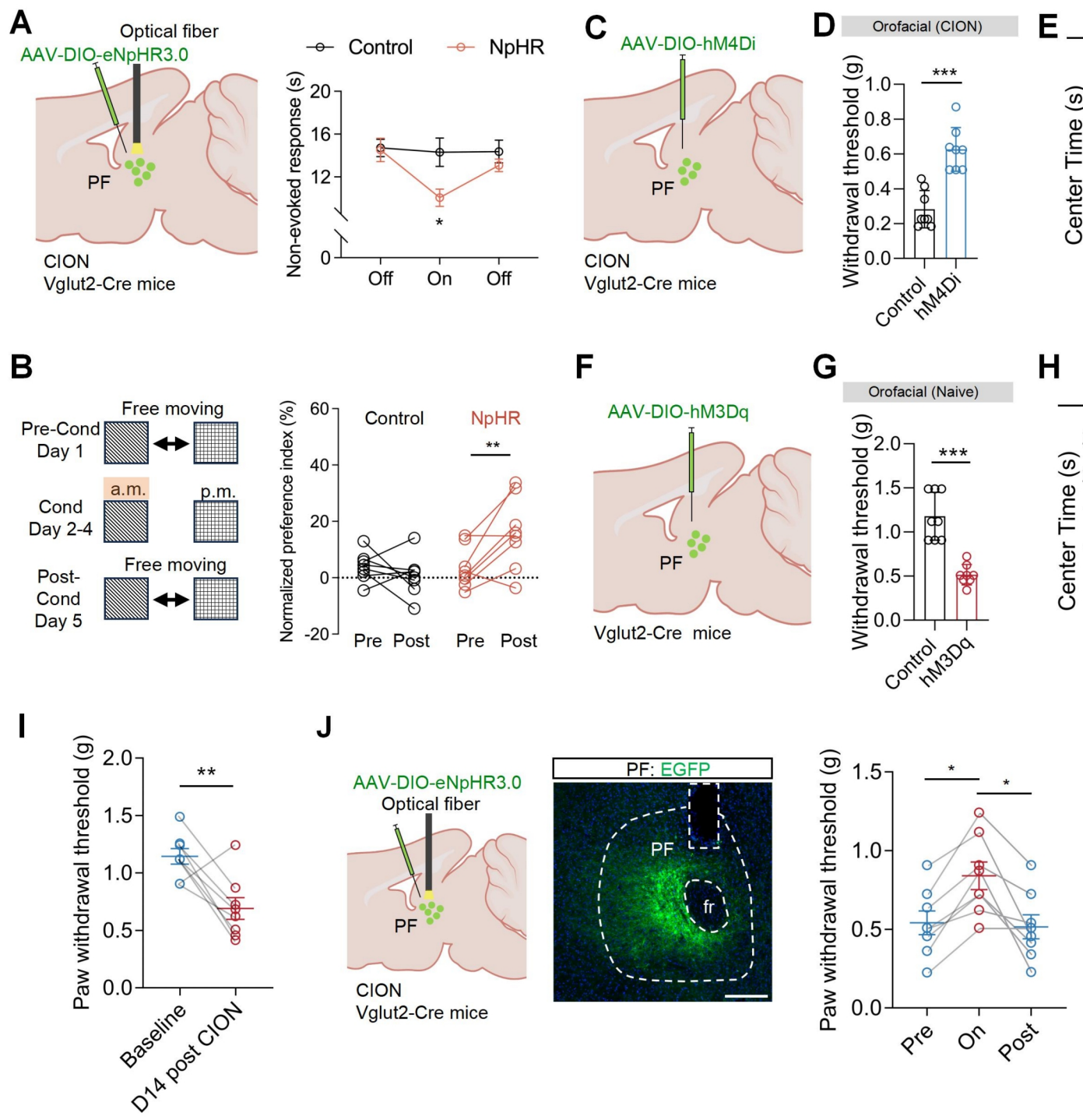

Supplemental Figure 2. Modulation of PF neurons is necessary and sufficient for sensory hypersensitivity and affective disorders in TN mice.

A, Schematic of virus injection and non-evoked responses of CION mice with 3 min light-off, 3 min light-on, and 3 min light-off. **B**, Procedure and normalized preference index of CPP performance (*n* = 8 mice for each group). **C,** Schematic of virus injection. **D**, Withdrawal threshold in von Frey test of CION mice between control and hM4Di. **E**, Behavioral performance of CION mice between control and hM4Di in the OFT and EPM test. **F**, Schematic of virus injection. **G**, Withdrawal threshold in von Frey test of naive mice between control and hM3Dq. **H**, Behavioral performance of naive mice between control and hM3Dq in the OFT and EPM test. **I**, Hindpaw withdrawal thresholds of mice before (Baseline) and 14 days post CION surgery. **J**, Effect of optogenetic inhibition of the PF on hindpaw withdrawal thresholds on day 14 post-CION. Data are presented as means ± s.e.m. Paired Student's *t*-test for **B and I**, Unpaired Student's *t*-test for **D**, **E**, **G**, **H**, One-way ANOVA with Tukey's post hoc test for **J**, Two-way ANOVA with Tukey's post hoc test for **A**. **P* < 0.05, ***P* < 0.01, ****P* < 0.001.

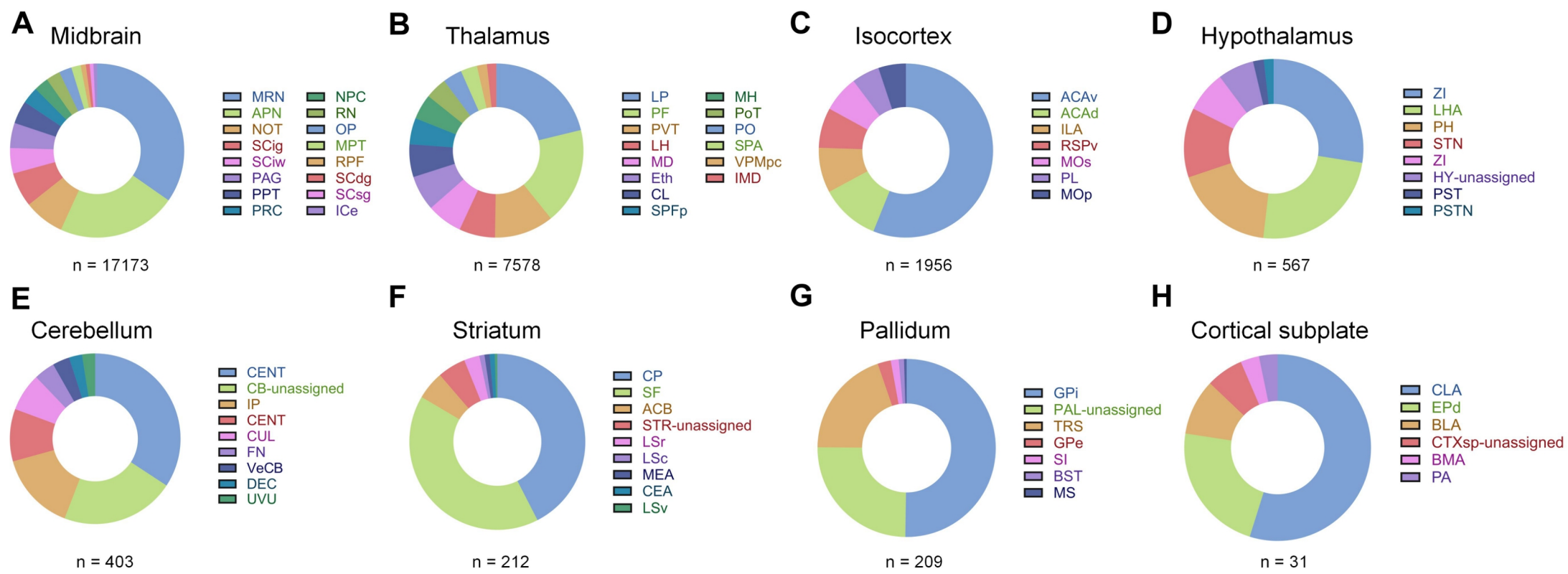

Supplemental Figure 3. Quantification of whole-brain transparency fluorescent-labeled cell somata in different upstream brain regions.

A, Midbrain. **B**, Thalamus. **C**, Isocortex. **D**, Hypothalamus. **E**, Cerebellum. **F**, Striatum. **G**, Pallidum. **H**, Cortical subplate.

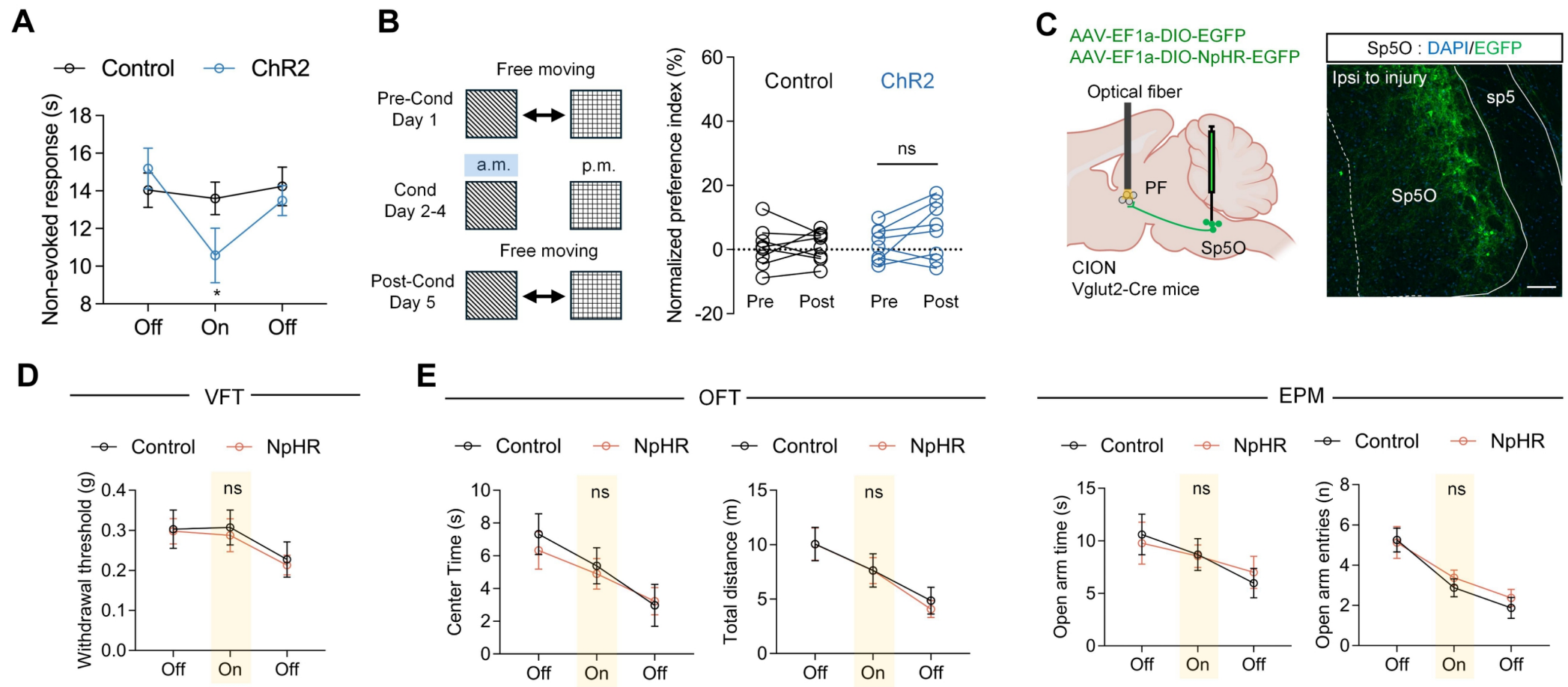

Supplemental Figure 4. Sp5O^{Glu}-PF circuit is not involved in orofacial pain and anxiety-like behavior regulation.

A, Non-evoked responses of CION mice with 3 min light-off, 3 min light-on, and 3 min light-off. **B**, Procedure and normalized preference index of CPP performance (n = 8 mice for each group). **C-E**, Schematic of virus injection and a representative image of EGFP expression at the Sp5O injection site (**C**), behavioral performance of mice with CION in the VFT (**D**), the OFT and the EPM (**E**) with 3 min light-off, 3 min light-on, and 3 min light-off (n = 8 mice for each group). Data are presented as means ± s.e.m. Paired Student's t-test for **B**, Two-way ANOVA with Tukey's post hoc test for **A**, **D** and **E**; ns, not significant.

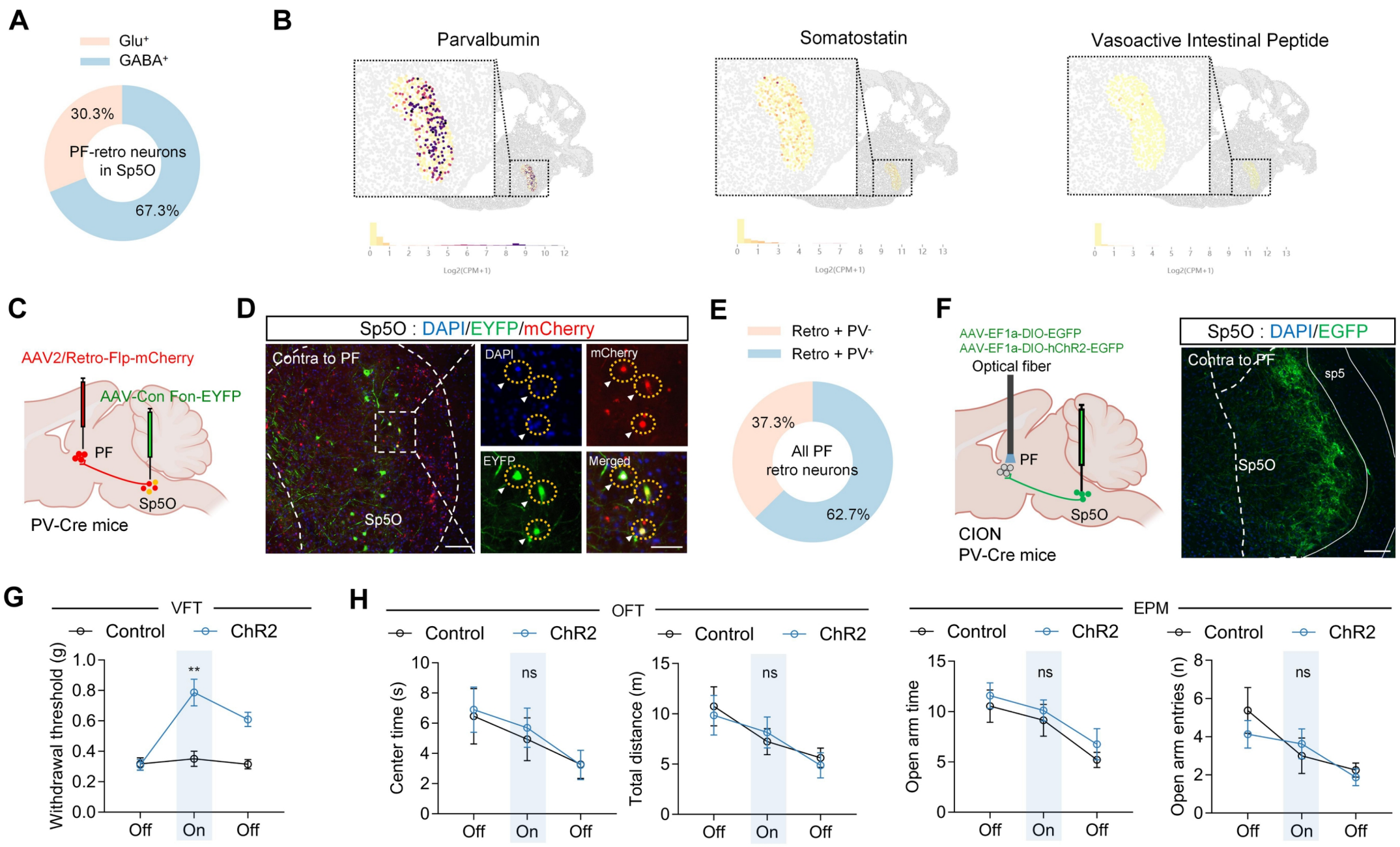

Supplemental Figure 5. Sp5O^{PV} neurons drive sensory but not affective dimensions of trigeminal neuralgia.

A, statistics on the proportion of GABAergic and glutamatergic neurons in the Sp5O that project to the PF. **B**, Representative Allen Brain Cell Atlas of parvalbumin, somatostatin, and vasoactive intestinal peptide expression in Sp5O. **C-E**, Schematic of virus injection (**C**), representative EYFP images in the Sp5O (**D**), and statistics on the proportion of PV neurons in the Sp5O that project to the PF (**E**) (scale bar, 50 μm; zoomed image, 20 μm). **F**, Schematic of virus injection and a representative image showing EGFP expression at the Sp5O injection site. **G-H**, behavioral performance of CION mice in the von Frey test (**G**), OFT and EPM (**H**) with 3 min light-off, 3 min light-on, and 3 min light-off (n = 8 mice for each group). Data are presented as means ± s.e.m. Two-way ANOVA with Tukey's post hoc test for **G**, **H**; $^{**}P < 0.01$; ns, not significant.

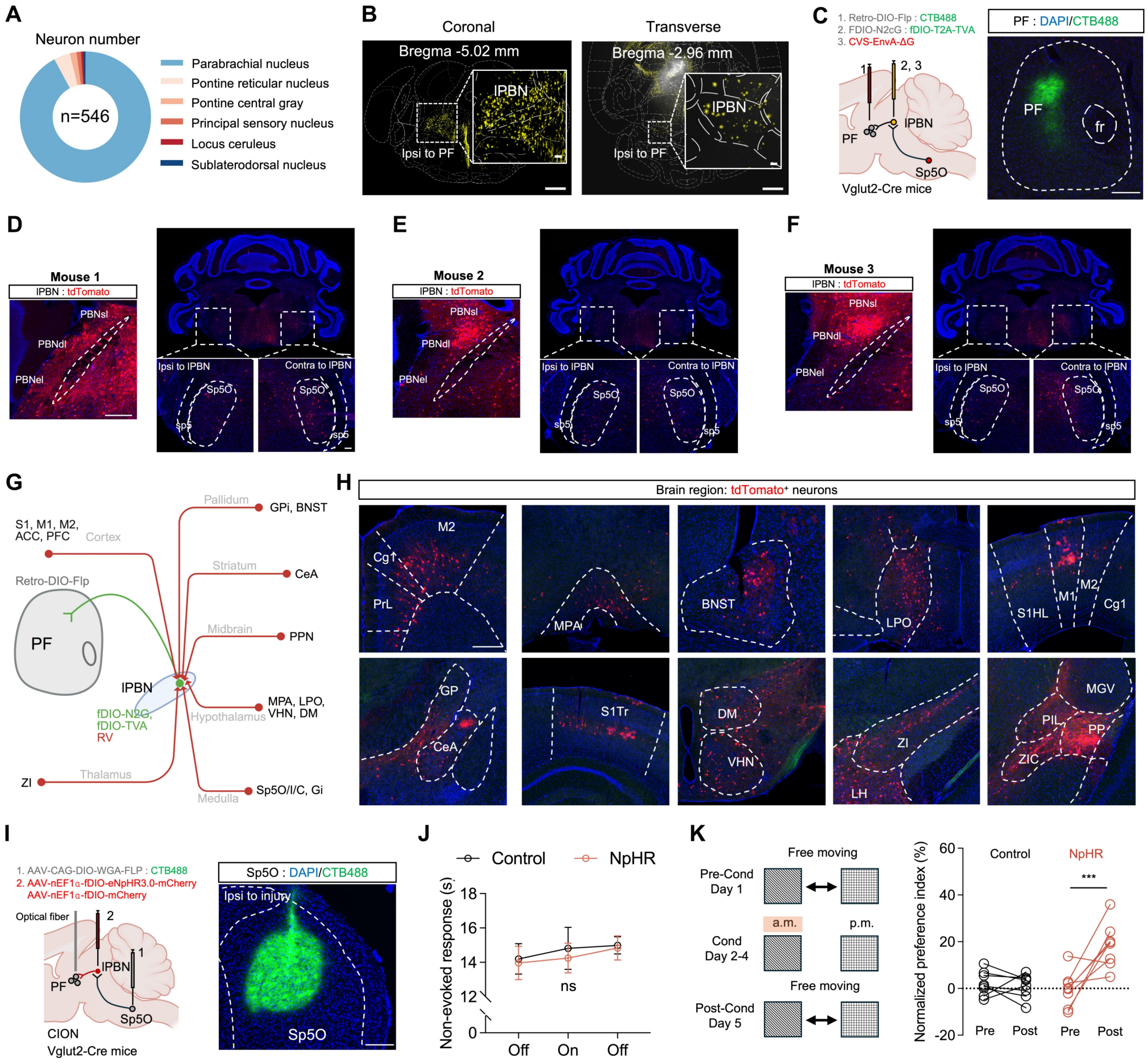

Supplemental Figure 6. Multi-regional projections from upstream brain areas to the PF via lPBN.

A, Quantification of whole-brain transparency fluorescent-labeled cell somata in the Pons. **B**, Coronal and transverse sections showing lPBN fluorescent-labeled cell somata (scale bar, 200 μm). **C**, Schematic of virus injection and representative images of CTB488 in PF (scale bar, 200 μm). **D-F,** Images of tdTomato expression in lPBN and Sp5O (scale bar, 200 μm) (The left image in Supplemental Figure 6D is reproduced from Figure 3H and is included here as part of the complete dataset from three individual mice). **G-H,** Summary schematic of viral-based retrograde anatomy results of lPBN neurons that project to the PF (**G**) and representative images of tdTomato expression in selected brain regions (scale bar, 200 μm) (**H**). **I,** Schematic of virus injection and representative images of CTB488 in Sp5O (scale bar, 50 μm) injection sites. **J**, Non-evoked responses of CION mice with 3 min light-off, 3 min light-on, and 3 min light-off. **K**, Procedure and normalized preference index of CPP performance (n = 8 mice for each group). Data are presented as means ± s.e.m. Paired Student's t-test for **K** and two-way ANOVA with Tukey's post hoc test for **J**; ***P < 0.001; ns, not significant.

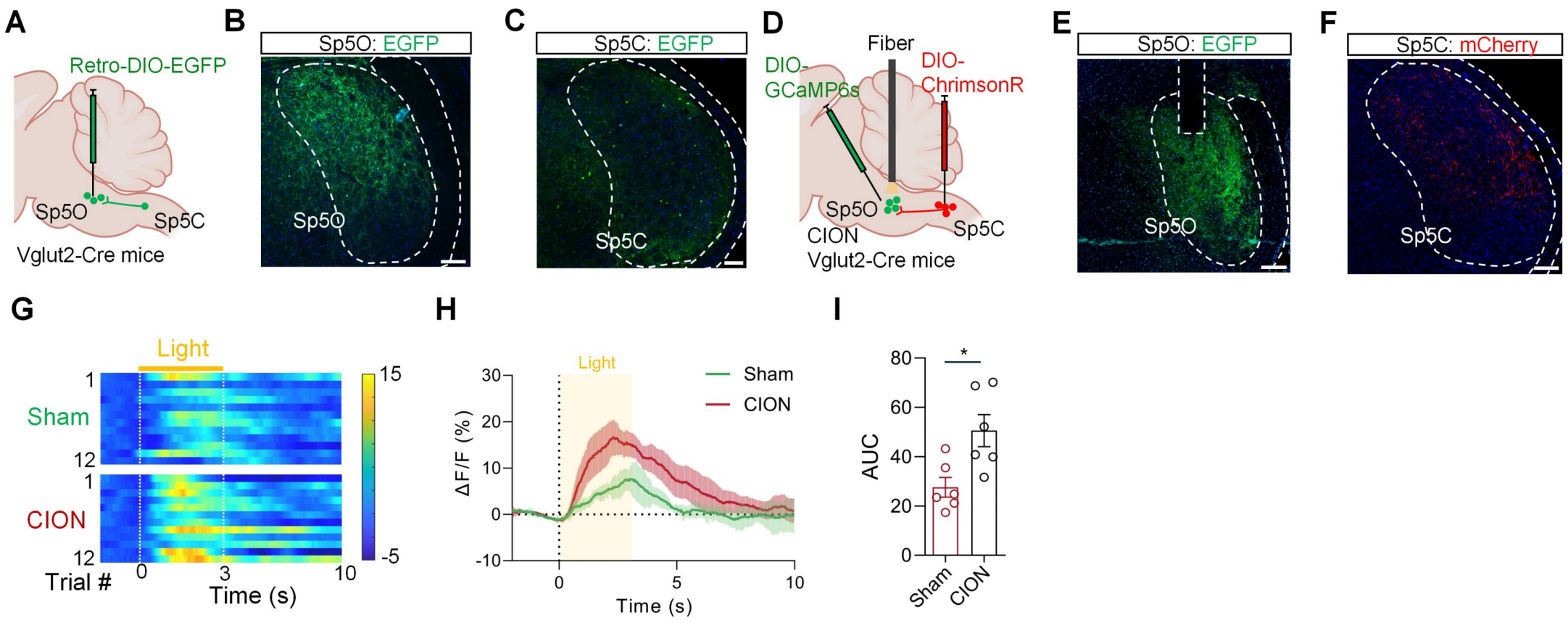

Supplemental Figure 7. Synaptic connectivity of the Sp5C→Sp5O projection is pathologically potentiated following CION. A, Schematic of virus injection. **B** and **C**, Representative images of EGFP in Sp5O (**B**) and Sp5C (**C**) (scale bar, 50 μm). **D**, Schematic of virus injection. **E** and **F**, Representative images of GCaMP6s in Sp5O (**E**) and mCherry in Sp5C (**F**) (scale bar, 50 μm). **G-I**, heatmaps (**G**), and averaged normalized traces (**H**) and quantification (area under the curves, AUC) of GCaMP6s signals (**I**) (Sham/CION, n = 12 trials from six mice). Data are presented as means ± s.e.m. Unpaired Student's t-test for I; *P < 0.5.

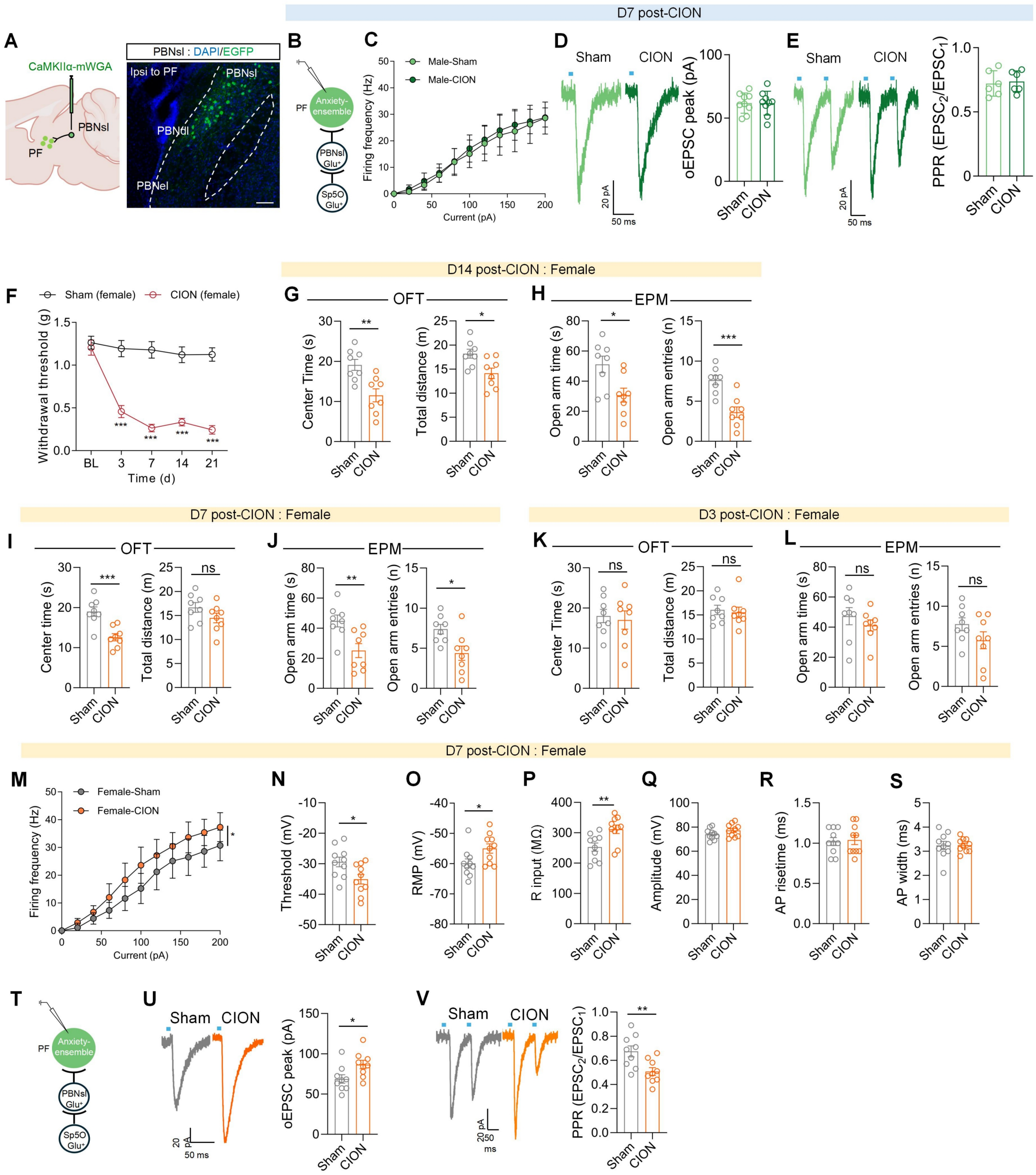

Supplemental Figure 8. Characterization of behavioral effects and PF anxiety-ensemble hyperexcitability in CION male and female mice.

A, Schematic of virus injection and representative images of EGFP in PBNsl injection sites (scale bar, 200 μm). **B**, Schematic of patch clamp on PF neurons. **C**, Firing frequency of APs in PF anxiety-ensemble neurons of male mice on day 7 after surgery (n = 10 cells from three mice). **D**, Example traces and peak amplitude of blue light (5 ms, 2 mW)-evoked oEPSCs in PF anxiety-ensemble neurons on day 7 after surgery. **E**, Example traces and PPR of blue light (5 ms, 2 mW, 100 ms interval)-evoked oEPSCs recorded in PF anxiety ensemble neurons from male mice. **F**, Behavioral performance of female mice with Sham and CION in the von Frey test. **G-L**, Behavioral performance of mice in the OFT, and EPM on day 14 (G and H), 7 (I and J), 3 (K and L) after surgery. **M**, CION modeling increases the firing frequency of APs evoked by depolarizing current in PF anxiety-ensemble neurons of female mice on day 7 after modeling (n =

12 cells from three mice). **N-S**, AP firing threshold (**N**), RMP (**O**), input resistance (**P**), AP firing amplitude (**Q**), risetime (**R**) and width (**S**) in Sham and CION PF anxiety-ensemble neurons of female mice on day 7 after modeling. **T**, Schematic of patch clamp. **U**, Example traces and peak amplitude of blue light (5 ms, 2 mW)-evoked oEPSCs in PF anxiety-ensemble neurons from Sham and CION female mice. **V**, Example traces and PPR of blue light (5 ms, 2 mW, 100 ms interval)-evoked oEPSCs recorded in PF anxiety-ensemble neurons from Sham and CION female mice. Data are presented as means ± s.e.m. Unpaired Student's t-test for **D**, **E**, **G-L**, **N-S**, **U** and **V**, Two-way ANOVA with Tukey's post hoc test for **C**, **F** and **M**; *P < 0.05, **P < 0.01, ***P < 0.001.

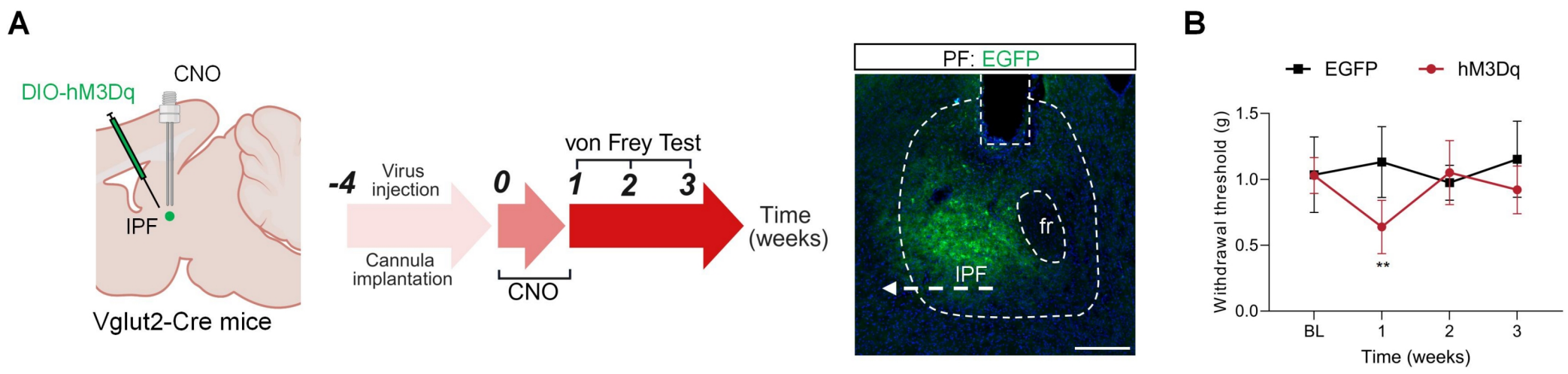

Supplemental Figure 9. Continuous activation of the lPF is insufficient to induce a sustained decrease in facial pain thresholds in mice. **A**, Schematic of virus injection and experiment and representative images of EGFP in PF (scale bar, 200 μm). **B**, Statistics of paw withdrawal threshold in the chemogenetic excitatory group and control mice during and/or after daily CNO administration for one week. Data are presented as means ± s.e.m. Two-way ANOVA with Tukey's post hoc test for **B**; **P < 0.01.

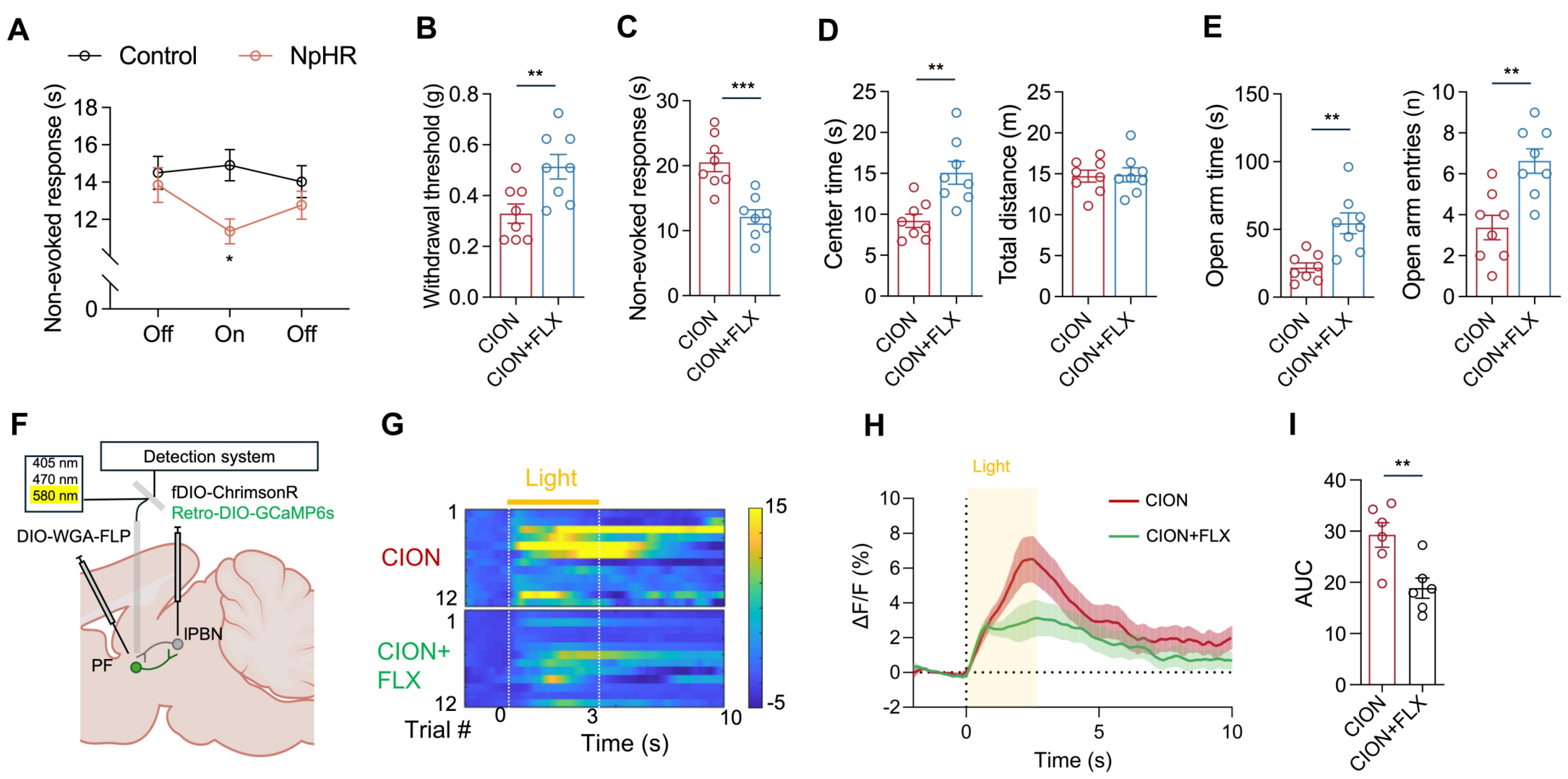

Supplemental Figure 10. Effects of SSRI treatment on TN-related behaviors and lPBN-mPF-lPBN loop activity.

A, Non-evoked responses of CION mice with 3 min light-off, 3 min light-on, and 3 min light-off. **B-E**, Behavioral performance of mice in the von Frey test (**B**), non-evoked response (**C**), OFT (**D**), and EPM (**E**). **F-I**, Schematic (**F**), heatmaps (**G**), and averaged

normalized traces (**H**) and quantification (area under the curves, AUC) of GCaMP6s signals (**I**) (CION/CION+FLX, n = 12 trials from six mice). Data are presented as means ± s.e.m. Unpaired Student's t-test for **B**-**E**, **I** and two-way ANOVA with Tukey's post hoc test for **A**; $*P < 0.5$, $**P < 0.01$, $***P < 0.001$.

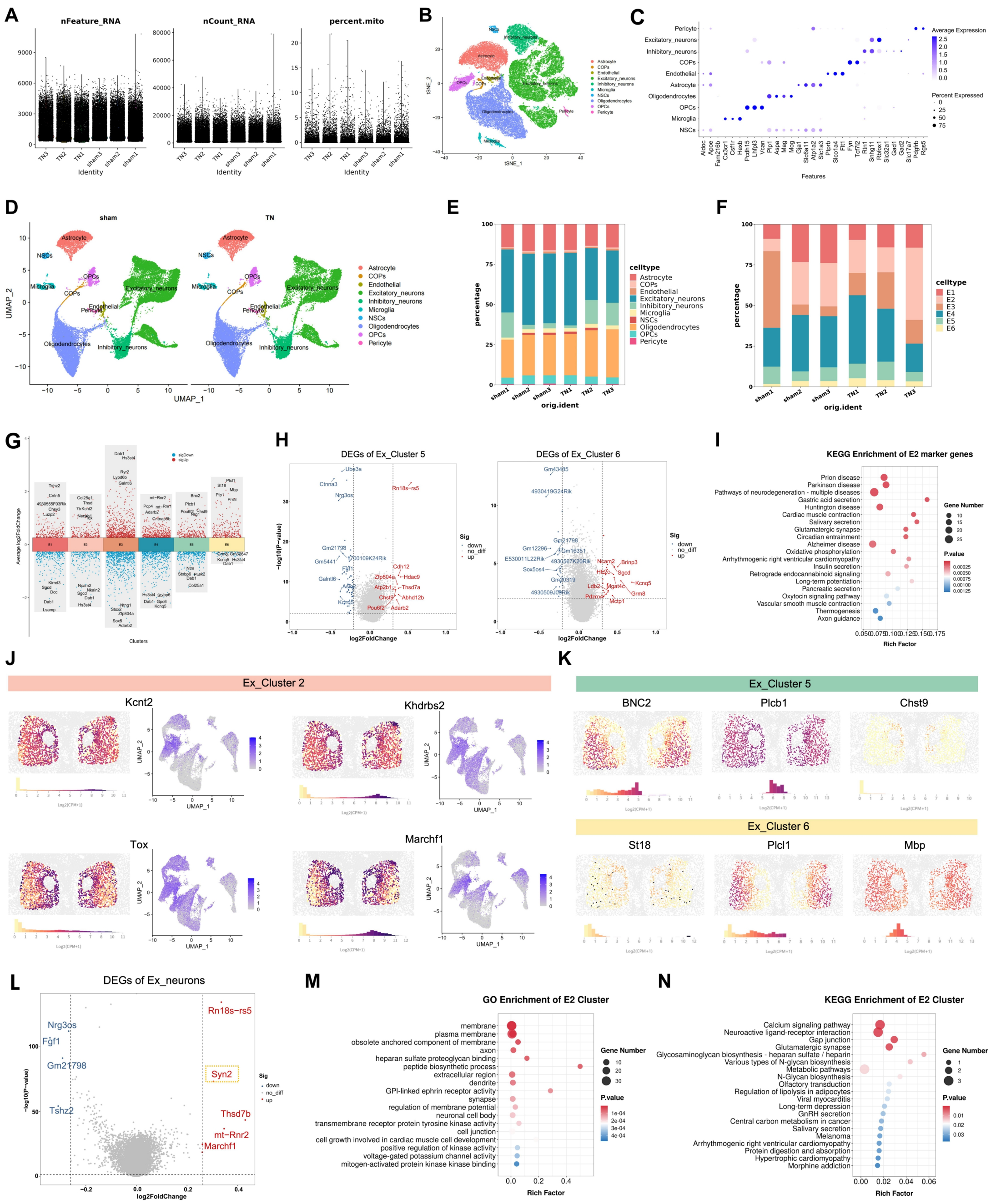

Supplemental Figure 11. Additional information for snRNA-seq experiments.
A, Distribution of cell nuclei in each sample after cell nucleus filtration. **B**, t-SNE dimensional reduction and visualization of transcriptional profiles of all cell types in the PF. **C**, Dot plot showing scaled expression levels (color) and the proportions of expressing cells (dot size) of marker genes in individual cell types. **D**, UMAP dimensional reduction and visualization of transcriptional profiles of all cell types in

the PF of Sham and CION groups. **E-F**, Proportions of different subpopulations of cells for all cell types (**E**) and only excitatory neurons (**F**) in the PF. **G**, Volcano plots showing expression levels of marker genes in individual PF excitatory neuron clusters. **H**, Differentially expressed genes (DEGs) in E5 and E6 Clusters between Sham and CION. **I**, KEGG enrichment scatter plot of E2 Cluster marker genes. **J**, Feature plots showing the expression patterns of marker genes (*Kcnt2*, *Khdrbs2*, *Tox* and *Marchf1*) of E2 Cluster and representative Allen Brain Cell Atlas in the PF. **K**, Representative Allen Brain Cell Atlas of the top three marker genes of E5 and E6 Clusters in the PF. **L**, DEGs of all excitatory neurons in the PF between Sham and CION groups. **M-N**, GO (**M**) and KEGG (**N**) enrichment bar plots of DEGs in E2 Cluster.

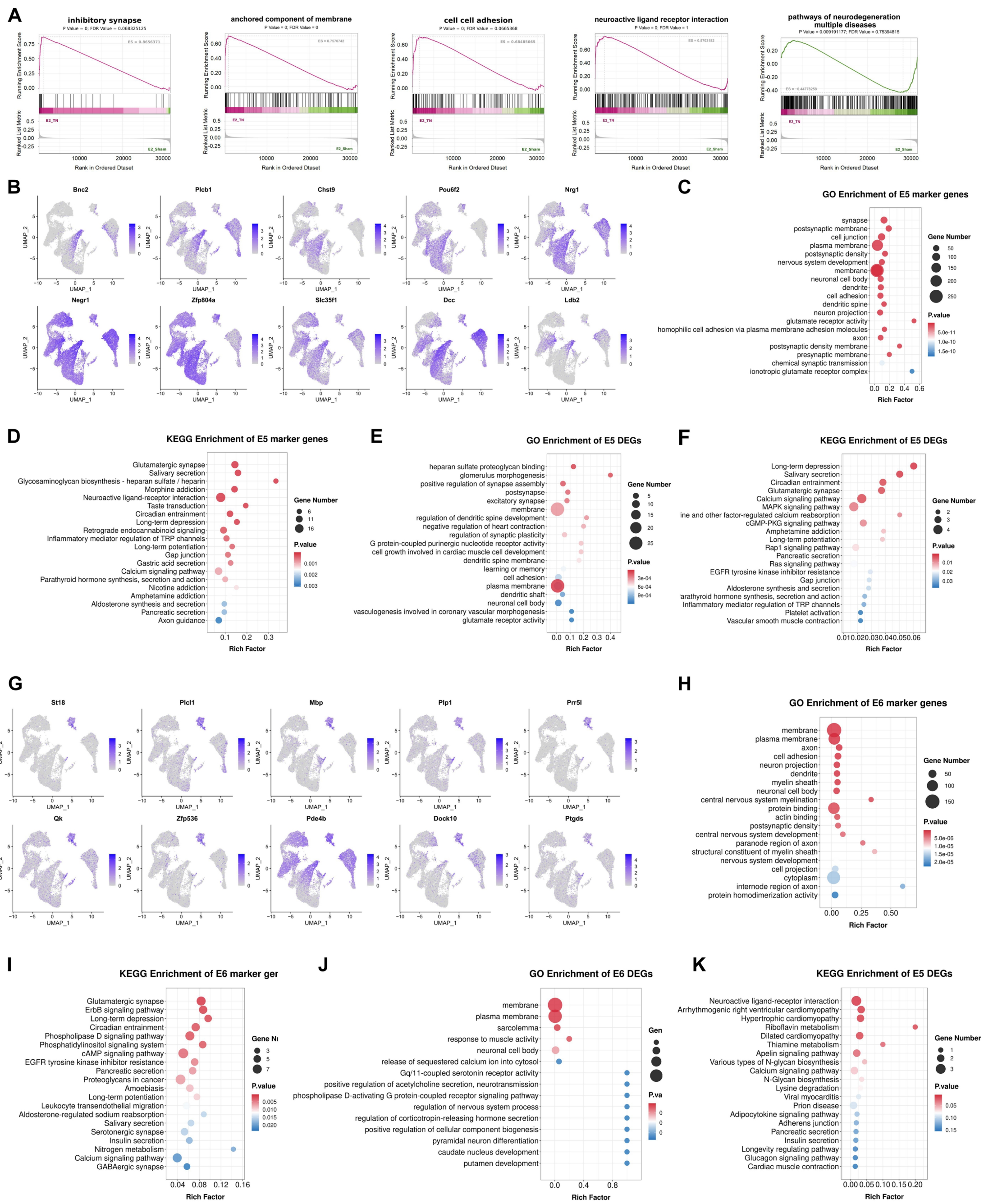

Supplemental Figure 12. Characterization of E5 and E6 clusters.

A, Gene set enrichment analysis (GSEA) for differentially expressed genes of the E2 cluster in Sham and CION groups, showing upregulation in the function of inhibitory synapse, anchored component of membrane, cell adhesion, and neuroactive ligand receptor interaction, while downregulation in pathways of neurodegeneration (multiple diseases). **B**, Feature plots showing the expression patterns of the top ten marker genes of E5 Cluster. **C**-**D**, GO and KEGG enrichment scatter plots of marker genes of E5 Cluster. **E**-**F**, GO and KEGG enrichment scatter plots of DEGs of E5 Cluster (CION vs Sham). **G**, Feature plots showing the expression patterns of the top ten marker genes of E6 Cluster. **H**-**I**, GO and KEGG enrichment scatter plots of marker genes of E6 Cluster. **J**-**K**, GO and KEGG enrichment scatter plots of DEGs of E6 Cluster (CION vs Sham).

Supplemental References

1. Iglesias JE, et al. A probabilistic atlas of the human thalamic nuclei combining ex vivo MRI and histology. *Neuroimage*. 2018;183:314–326.
2. Sheng H-Y, et al. Activation of ventrolateral orbital cortex improves mouse neuropathic pain–induced anxiodepression. *JCI Insight*;5(19):e133625.
3. Boccella S, et al. Spared Nerve Injury as a Long-Lasting Model of Neuropathic Pain. In: Skaper SD, ed. *Neurotrophic Factors*. New York, NY: Springer New York; 2018:373–378.
4. Chaplan SR, et al. Quantitative assessment of tactile allodynia in the rat paw. *J Neurosci Methods*. 1994;53(1):55–63.
5. Xu M, Aita M, Chavkin C. Partial infraorbital nerve ligation as a model of trigeminal nerve injury in the mouse: Behavioral, neural, and glial reactions. *The Journal of Pain*. 2008;9(11):1036–1048.
6. Vos BP, Strassman AM, Maciewicz RJ. Behavioral evidence of trigeminal neuropathic pain following chronic constriction injury to the rat's infraorbital nerve. *J Neurosci*. 1994;14(5 Pt 1):2708–2723.
7. Slyper M, et al. A single-cell and single-nucleus RNA-seq toolbox for fresh and frozen human tumors. *Nat Med*. 2020;26(5):792–802.
8. Butler A, et al. Integrating single-cell transcriptomic data across different conditions, technologies, and species. *Nat Biotechnol*. 2018;36(5):411–420.
9. McGinnis CS, Murrow LM, Gartner ZJ. DoubletFinder: Doublet detection in single-cell RNA sequencing data using artificial nearest neighbors. *Cell Syst*. 2019;8(4):329-337.e4.
10. Stuart T, et al. Comprehensive integration of single-cell data. *Cell*. 2019;177(7):1888-1902.e21.
